# A Systematic Review and Meta-Analysis on Multiple Cytokine Gene Polymorphisms in the Pathogenesis of Periodontitis

**DOI:** 10.3389/fimmu.2021.713198

**Published:** 2022-01-03

**Authors:** Xin Liu, Hui Li

**Affiliations:** Department of Stomatology, China-Japan Union Hospital of Jilin University, Changchun, China

**Keywords:** meta-analysis, periodontitis, T helper cell, interleukin-1, genetic polymorphism

## Abstract

**Aim:**

Periodontitis is an inflammatory disease that destroys both soft and hard periodontal tissues. However, a complex periodontal cytokine network remains unclear. This systematic review explored multiple cytokine gene polymorphisms in the pathogenesis of periodontitis.

**Material and Methods:**

A systematic search was performed using the databases from previous publications, which indicated the association between cytokine polymorphisms and periodontitis pathogenesis. Meta-analysis was conducted using fixed or randomized models to calculate the significance of multiple cytokine polymorphisms. A total of 147 articles were analyzed with polymorphisms in 12 interleukins [Th1 (IL-2, IFN-γ, and TNF-α), Th2 (IL-4 and IL-13), Th17 (IL-1α, IL-1β, IL-6, and IL-17), and Treg cytokines (IL-10 and TGF-β)]. Doi plot was used to probe the occurrence of publication bias.

**Results:**

The polymorphisms of IL-2 and TNF-α of Th1 cytokine family may be associated with the pathogenesis or the prevention of periodontitis risk, while the polymorphism of IFN-γ is not related to periodontitis risk. The polymorphisms for IL-4 and IL-13 of Th2 cytokine family are not found to be associated with the pathogenesis of periodontitis. For the polymorphisms of the members of Th17 cytokine family, different IL-1α polymorphisms may have inverse actions in the pathogenesis of periodontitis. IL-1β is a noteworthy cytokine biomarker in periodontitis development and progression. IL-6 may have a protective function in the inflammatory responses of periodontitis, and IL-17 has a weak relationship the inflammatory responses. The polymorphisms for the members of Treg cell cytokines may have a protective function against periodontitis risk. LFK indexes show the major asymmetry due to publication bias.

**Conclusion:**

IL-1β is a notable cytokine biomarker in periodontitis risk. Treg cytokines favor an anti-inflammatory and protective environment. Further data are needed to confirm the present conclusion due to publication bias.

## Introduction

Periodontitis is a chronic destructive inflammatory disease that destroys the tooth-supporting structures and results in tooth loss (1). The cytokine network plays an important role in periodontitis development and immune responses (2), and its association with periodontitis remains widely unclear. Cytokine expression profiles are closely related to Th1, Th2, Th17, and Treg cells, which play an immunoregulatory role (3). Interferon‐γ (IFN-γ), tumor necrosis factor α (TNF-α), and IL-2 belong to Th1 cytokines (4); and T follicular helper cells have been shown to be a significant source of IL-4 and IL-13 in the lymph nodes during the Th2 activity (5). Th17 cells are the major source of interleukin-17 (6), TGF-β (7), IL-1 and IL-6 (8), and IL-23 (9). Anti-inflammatory IL-10 (10) and TGF-β (11) are secreted by B cell-endowed Tregs, involved in the suppressive function of regulatory T cells, and play critical roles in maintaining immune homeostasis.

Many studies have proven gene polymorphisms in cytokines such as IL‐1α (12–28), IL-1β (12, 16, 18, 24, 29–55), IL-2 (56–60), IL-4 (61–73), IL‐6 (74–86), IL-23 (87), IL-10 (16, 42, 84–86, 88–110), IL-13 (66, 111, 112), IL-17 (60, 73, 87, 113–117), TNF-α (16, 35, 38, 44, 48, 85, 118–132), IFN (70, 85, 102, 133–136), and TGF (42, 137–143), which often play various vital roles in periodontitis immune pathogenesis or in the prevention of periodontitis immune responses.

However, the genetic polymorphisms of these cytokines were often analyzed separately and seldom compared together. The significant differences remain unclear. Therefore, in the current meta‐analysis, a total of 147 studies were included to further identify the contributions of these genetic polymorphisms using a broader collection of patients and races.

## Materials and Methods

### Search Strategy and Inclusion Criteria

The criteria for inclusion in our meta‐analysis were as follows: 1) case–control studies; 2) the case groups consisted of patients diagnosed with periodontitis, and the control groups consisted of periodontally healthy individuals; 3) the genetic polymorphisms were detected, and sufficient data supporting the genotype distribution were provided for the calculation of odds ratios (ORs) and corresponding 95% confidence intervals (CIs); and 4) studies with no repeated data. Studies that did not fit any of the criteria were excluded.

A systematic literature search was performed using the databases PubMed, the Cochrane Library, Medical Abstracts, TOXLINE, OVID, EMBASE, Web of Science, EBSCO, VIP Full Text, CNKI, and Wanfang, including studies published up to January 31, 2021. In addition, the reference lists of the selected manuscripts and related reviews were also manually searched and screened to ensure that a comprehensive search was performed. Furthermore, no language restriction was applied. Two authors independently searched all the databases. The following key terms were used for searching; “interleukin‐1”, “interleukin 1”, “IL‐1”, “IL1”, “interleukin‐2”, “interleukin 2”, “IL‐2”, “IL2”, “interleukin‐4”, “interleukin 4”, “IL‐4”, “IL4”, “interleukin‐6”, “interleukin 6”, “IL‐6”, “IL6”, “interleukin-10”, “interleukin 10”, “IL‐10”, “IL10”, “interleukin‐13”, “interleukin 13”, “IL‐13”, “IL13”, “interleukin‐17”, “interleukin 17”, “IL‐17”, “IL17”, “interferon‐gamma”, “interferon gamma”, “IFN-gamma”, “IFN gamma”, “interferon‐γ”, “interferon γ”, “IFN-γ”, “IFN γ”, “tumor necrosis factor‐alpha”, “tumor necrosis factor alpha”, “TNF‐alpha”, “TNF alpha”, “tumor necrosis factor‐α”, “tumor necrosis factor α”, “TNF‐α”, “TNF α”, “transforming growth factor-beta”, “transforming growth factor beta”, “TGF‐beta”, “TGF beta”, “transforming growth factor-β”, “transforming growth factor β”, “TGF‐β”, “TGF β”, “polymorphism”, “periodontal diseases”, “periodontitis”, and their combined phrase.

### Study Selection and Data Extraction

**Figure 1:**
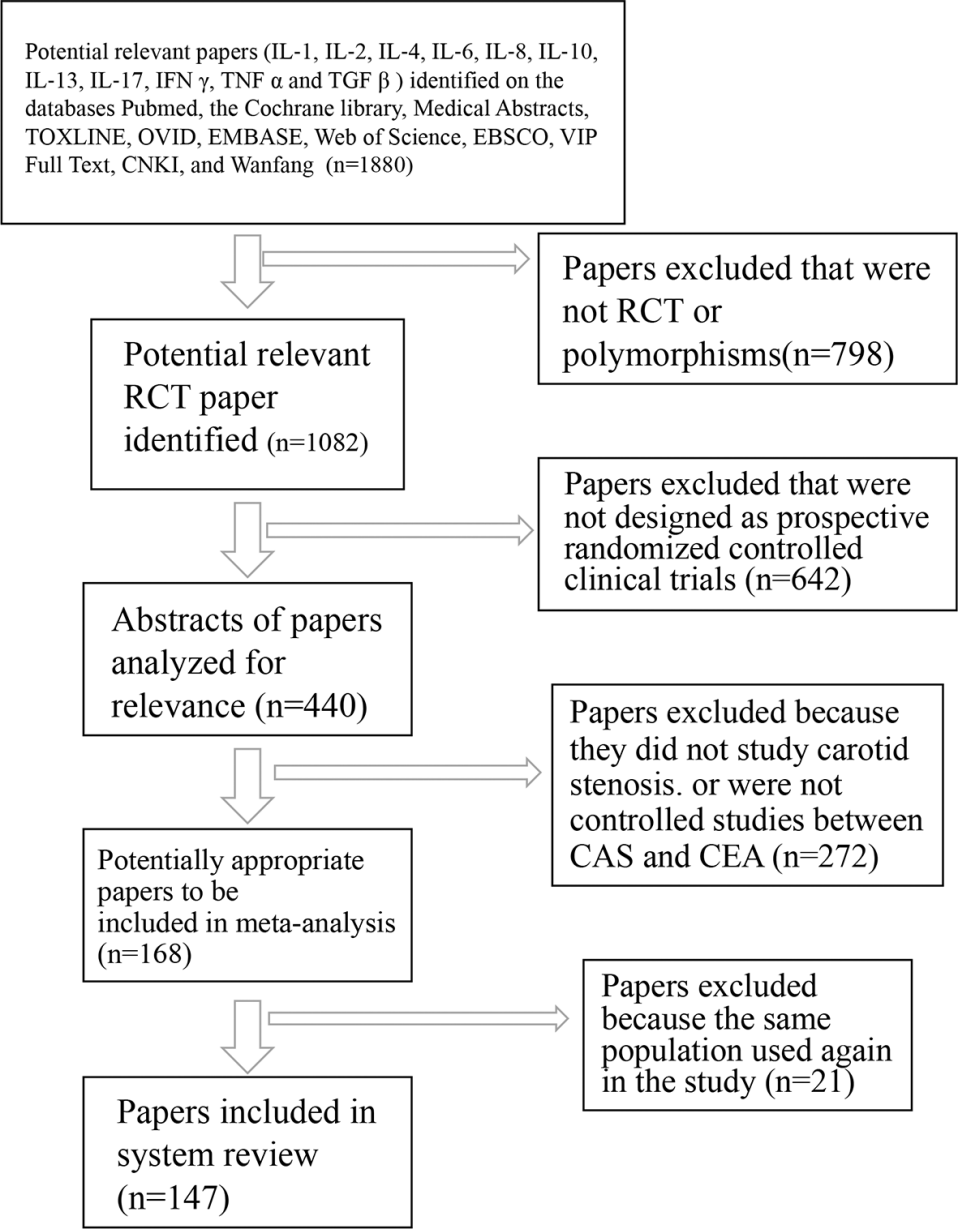
Flowchart of trial search and selection for meta-analysis. RCT, randomized and controlled trial; IL‐1, interleukin‐1; IL‐2, interleukin-2; IL‐4, interleukin‐4; IL‐6, interleukin‐6; IL‐8, interleukin‐8; IL‐10, interleukin-10; IL‐13, interleukin‐13; IL‐17, interleukin‐17; IFN-γ, interferon gamma; TNF-α, tumor necrosis factor alpha; TGF-β, transforming growth factor beta.

During the data selection, duplicate researches were removed using Endnote. The abstracts of selected papers were further screened, and the full text was checked based on the selection criteria (**Figure 1**). The results were examined by two authors, and an expert was referred if inconsistencies appeared. Two authors obtained the following characteristics, and inconsistencies were determined *via* discussion: 1) the first author name and publication years; 2) nationality and/or ethnicity; 3) population size; 4) genetic polymorphism; and 5) Hardy–Weinberg equilibrium (HWE) in these populations.

### Data Analysis

ORs and 95% CIs were analyzed to evaluate the relationship between genetic polymorphisms in 12 interleukins [Th1 (IL-2, IFN-γ, and TNF-α), Th2 (IL-4 and IL-13), Th17 (IL-1 α, IL-1β, IL-6, TGF-β, and IL-17), and Treg cytokines (IL-10 and TGF-β)] and periodontitis. The interstudy heterogeneity was estimated using I^2^, where I^2^ > 50% and p < 0.05 were regarded as significant heterogeneity. Subsequently, the random-effects model (Mantel–Haenszel method) was performed in this study. I^2^ < 50% and p > 0.05, and the fixed‐effects model (Mantel–Haenszel method) was performed to confirm collective effectiveness. One or all the following genetic polymorphisms were analyzed: 1) genetic specific allele; 2) dominant model; and/or 3) recessive model. Statistical analyses were carried out using MetaXL (https://www.epigear.com/index_files/metaxl.html).

### Publication Bias

Publication bias is a major problem in meta-analysis, which affects the strength of the final conclusion and remains suboptimal, and impedes the cogency and explanation of meta-analysis discoveries. When bias occurs, it typically differentially affects studies manifesting as an association between precision and effect size, and photographic asymmetry of common funnel plots. However, the asymmetry is quantified using Egger’s regression, and the sensitivity of Egger’s regression is difficult to detect such asymmetry when the amounts of studies are limited. A new graphical method, the Doi plot, to visualize asymmetry using the LFK index can detect the asymmetry of study effects well. The LFK index also has a higher sensitivity than Egger’s regression (144). Therefore, publication bias was assessed using LFK index test proposed by Furuya-Kanamori et al. LFK index zero represents complete symmetry, and the limits of symmetry were set at ±1, and values beyond ±1 were inconsistent with symmetry.

## Results

### Study Selection

According to the study flowchart (**Figure 1**), a total of 147 articles were included in the final meta‐analysis on genetic polymorphism in the 12 cytokine genes (Th1 (IL-2, IFN-γ, and TNF-α), Th2 (IL-4 and IL-13), Th17 (IL-1α, IL-1β, IL-6, TGF-β, and IL-17), and Treg cytokines (IL-10 and TGF-β)), including IL‐1α (12–28), IL-1β (12, 16, 18, 24, 29–55), IL-2 (56–60), IL-4 (61–73), IL‐6 (74–85, 145), IL-10 (16, 42, 84–86, 88–107, 109, 110, 118, 146, 147), IL-13 (66, 111, 112), IL-17 (60, 73, 87, 113–117), TNF‐α (16, 35, 38, 44, 48, 85, 118–132), IFN (70, 85, 102, 133–136), and TGF (42, 137–143). These studies encompassed various periodontitis cases and healthy controls, which were involved in the analysis of the gene polymorphism and play important roles in promoting or preventing either chronic or aggressive periodontitis. We explored the relationship between the genetic polymorphism and the occurrence of periodontitis risk or prevention of periodontitis development using these studies (**Tables S1****–****S12**), in which the data were used to test the association of alleles and genotype with periodontitis risk.

### Th1 Cell Cytokines

**Figure 2:**
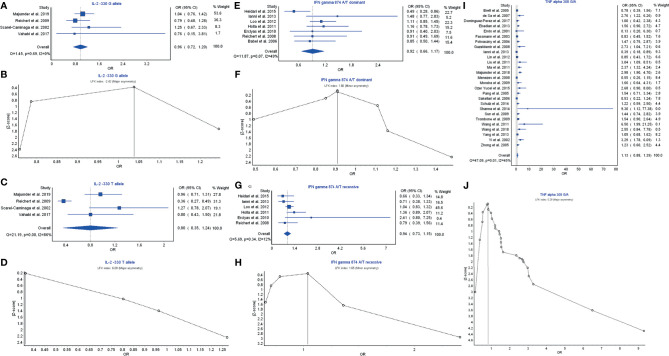
The association of Th1-related cytokine polymorphism with chronic periodontitis. **(A)** Forest plot of the association of IL‐2 −330 G allele polymorphism with chronic periodontitis. **(B)** The Doi plot is used to visualize the asymmetry of the association of IL‐2 −330 G allele polymorphism with chronic periodontitis using the LFK index. LFK index zero represents complete symmetry, the limits of symmetry were set at ±1, and values beyond ±1 were inconsistent with symmetry. **(C)** Forest plot of the association of IL‐2 −330 T allele polymorphism with chronic periodontitis. **(D)** The Doi plot is used to visualize the asymmetry of the association of IL‐2 −330 T allele polymorphism with chronic periodontitis using the LFK index. **(E)** Forest plot of the association of IFN-γ 874 A/T dominant polymorphism with chronic periodontitis. **(F)** The Doi plot is used to visualize the asymmetry of the association of IFN-γ 874 A/T dominant polymorphism with chronic periodontitis using the LFK index. LFK index zero represents complete symmetry, the limits of symmetry were set at ±1, and values beyond ±1 were inconsistent with symmetry. **(G)** Forest plot of the association of IFN-γ 874 A/T recessive polymorphism with chronic periodontitis. **(H)** The Doi plot is used to visualize the asymmetry of the association of IFN-γ 874 A/T recessive polymorphism with chronic periodontitis using the LFK index. **(I)** Forest plot of the association of TNF-α 308 G/A polymorphism with chronic periodontitis. **(J)** The Doi plot is used to visualize the asymmetry of the association of TNF-α 308 G/A polymorphism with chronic periodontitis using the LFK index. LFK index zero represents complete symmetry, the limits of symmetry were set at ±1, and values beyond ±1 were inconsistent with symmetry.

Th1 cytokines are structurally related to IL-2, IFN-γ, and TNF-α, whose polymorphisms are possibly associated with periodontitis risk. IL-2 −330G allele had a weak relationship with the periodontitis development with OR (95% CI), 0.96 (0.72–1.20) (**Figure 2A**). LFK index showed that there was major asymmetry and significant publication bias for the result of IL-2 −330G allele (**Figure 2B**). In contrast, IL-2 −330T allele had a strong relationship with the periodontitis risk with OR (95% CI), 0.80 (0.35–1.24) (**Figure 2C**). However, a higher level of LFK index also indicated that there was major asymmetry and publication bias for the result of IL-2 −330T allele (**Figure 2D**).

IFN-γ 874 A/T dominant model had a weak relationship with the periodontitis risk with OR (95% CI), 0.92 (0.66–1.17) (**Figure 2E**). LFK index showed that there was minor asymmetry and publication bias for the result of IFN-γ 874 A/T dominant model (**Figure 2F**). Similarly, IFN-γ 874 A/T recessive model also had a weak relationship with the periodontitis development with OR (95% CI), 0.94 (0.73–1.15) (**Figure 2G**). LFK index also indicated that there was minor asymmetry and publication bias for the result of IFN-γ 874 A/T recessive model (**Figure 2H**).

TNF-α 308 G/A polymorphism had a significant relationship in the prevention of periodontitis risk with OR (95% CI), 1.13 (0.88–1.39) (**Figure 2I**). LFK index showed that there was major asymmetry and significant publication bias for the result of TNF-α 308 G/A polymorphism (**Figure 2J**). Taken together, these results suggest that the members IL-2 and TNF-α of Th1 cytokine family may be associated with the pathogenesis of periodontitis or prevention of periodontitis risk, and their polymorphism can affect periodontitis risk while the polymorphism of IFN-γ is not associated with periodontitis risk.

### Th2 Cell Cytokines

**Figure 3:**
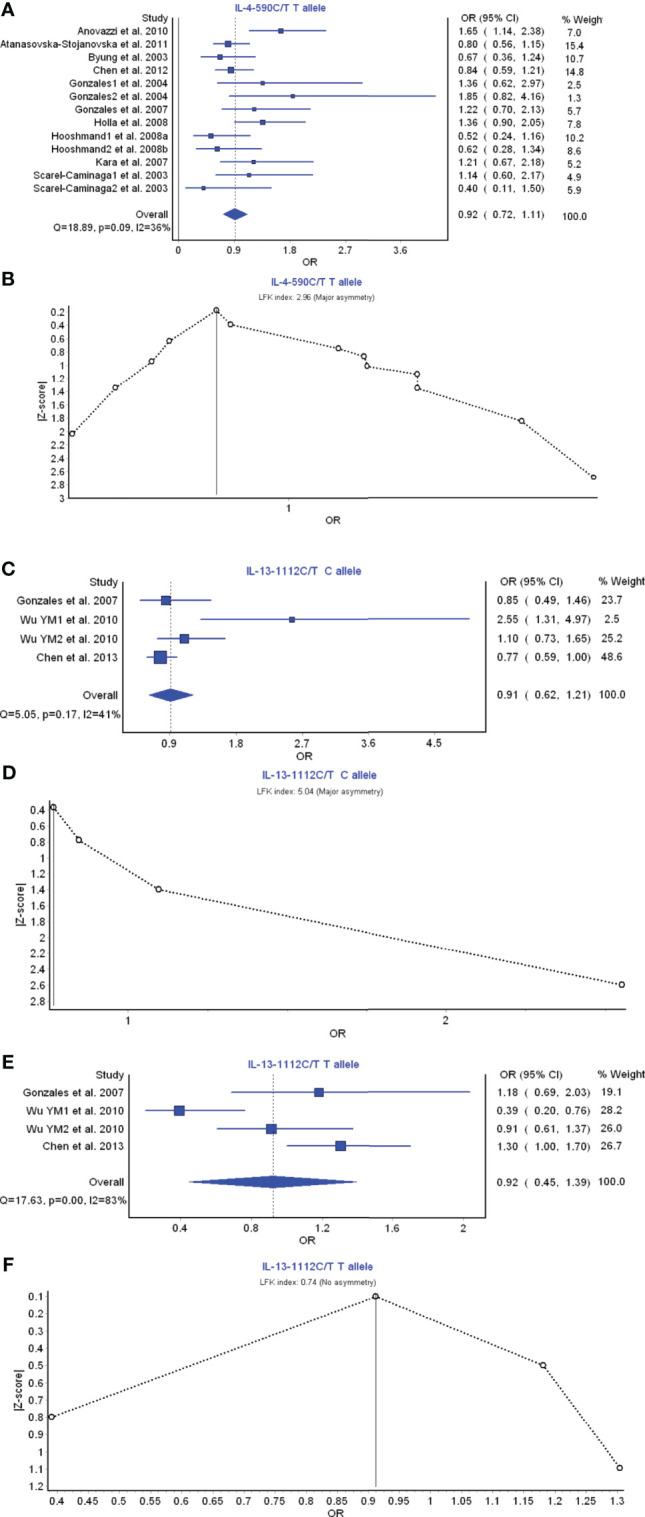
The association of Th2-related cytokine polymorphism with chronic periodontitis. **(A)** Forest plot of the association of IL-4 −590 C/T T allele polymorphism with chronic periodontitis. **(B)** The Doi plot is used to visualize the asymmetry of the association of IL-4 −590 C/T T allele polymorphism with chronic periodontitis using the LFK index. **(C)** Forest plot of the association of IL-13 −1112C/T C allele polymorphism with chronic periodontitis. **(D)** The Doi plot is used to visualize the asymmetry of the association of IL-13 −1112C/T C allele polymorphism with chronic periodontitis using the LFK index. **(E)** Forest plot of the association of IL-13 −1112C/T T allele polymorphism with chronic periodontitis. **(F)** The Doi plot is used to visualize the asymmetry of the association of IL-13 −1112C/T T allele polymorphism with chronic periodontitis using the LFK index. LFK index zero represents complete symmetry, the limits of symmetry were set at ±1, and values beyond ±1 were inconsistent with symmetry.

IL-4 and IL-13 are the members of Th2 cell cytokines. IL-4 −590 C/T T allele had a weak relationship with the periodontitis risk with OR (95% CI), 0.92 (0.72–1.11) (**Figure 3A**). LFK index showed that there was major asymmetry and significant publication bias for the result of IL-4 −590 C/T T allele (**Figure 3B**). IL-13 −1112C/T C allele had a weak relationship with the periodontitis risk with OR (95% CI), 0.91 (0.62–1.21) (**Figure 3C**). LFK index showed that there was major asymmetry and publication bias for the result of IL-13 −1112C/T C allele model (**Figure 3D**). Similarly, IL-13 −1112C/T T allele also had a weak relationship with the periodontitis development with OR (95% CI), 0.92 (0.45–1.39) (**Figure 3E**). LFK index indicated that there was no asymmetry or publication bias for the result of IL-13 −1112C/T T allele model (**Figure 3F**). Taken together, these results suggest that the members IL-4 and IL-13 of Th2 cytokine family may not be associated with the pathogenesis of periodontitis.

### Th17 Cell Cytokines

**Figure 4:**
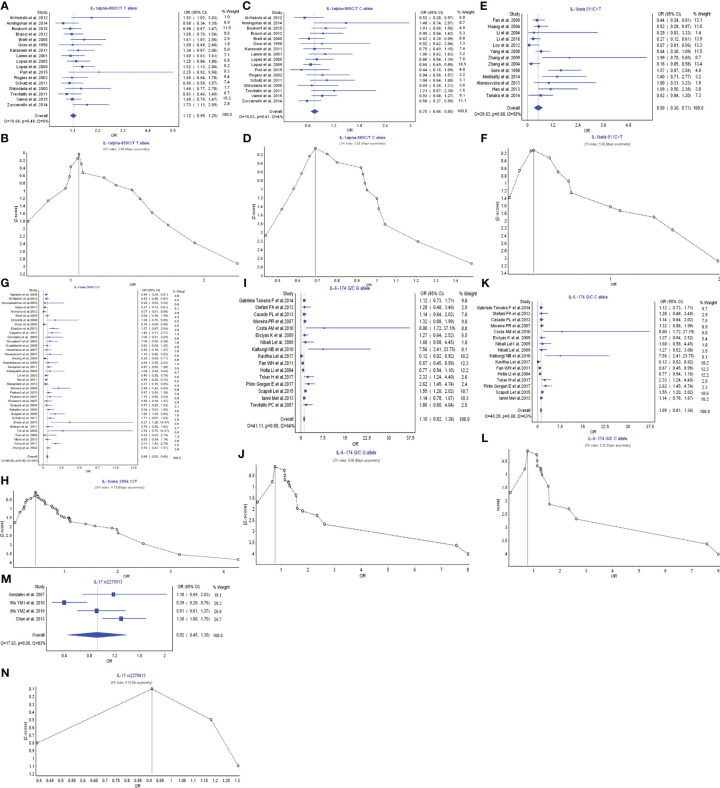
The association of Th17-related cytokine polymorphism with chronic periodontitis. **(A)** Forest plot of the association of IL-1α −889C/T T allele polymorphism with chronic periodontitis. **(B)** The Doi plot is used to visualize the asymmetry of the association of IL-1α −889C/T T allele polymorphism with chronic periodontitis using the LFK index. LFK index zero represents complete symmetry, the limits of symmetry were set at ±1, and values beyond ±1 were inconsistent with symmetry. **(C)** Forest plot of the association of IL-1α −889C/T C allele polymorphism with chronic periodontitis. **(D)** The Doi plot is used to visualize the asymmetry of the association of IL-1α −889C/T C allele polymorphism with chronic periodontitis using the LFK index. **(E)** Forest plot of the association of IL-1β −511C>T polymorphism with chronic periodontitis. **(F)** The Doi plot is used to visualize the asymmetry of the association of IL-1β −511C>T polymorphism with chronic periodontitis using the LFK index. LFK index zero represents complete symmetry, the limits of symmetry were set at ±1, and values beyond ±1 were inconsistent with symmetry. **(G)** Forest plot of the association of IL-1β 3954C>T polymorphism with chronic periodontitis. **(H)** The Doi plot is used to visualize the asymmetry of the association of IL-1β 3954C>T polymorphism with chronic periodontitis using the LFK index. **(I)** Forest plot of the association of IL-6 −174G/C G allele polymorphism with chronic periodontitis. **(J)** The Doi plot is used to visualize the asymmetry of the association of IL-6 −174G/C G allele polymorphism with chronic periodontitis using the LFK index. **(K)** Forest plot of the association of IL-6 −174G/C C allele polymorphism with chronic periodontitis. **(L)** The Doi plot is used to visualize the asymmetry of the association of IL-6 −174G/C C allele polymorphism with chronic periodontitis using the LFK index. **(M)** Forest plot of the association of IL-17 rs2275913 polymorphism with chronic periodontitis. **(N)** The Doi plot is used to visualize the asymmetry of the association of IL-17 rs2275913 polymorphism with chronic periodontitis using the LFK index. LFK index zero represents complete symmetry, the limits of symmetry were set at ±1, and values beyond ±1 were inconsistent with symmetry.

IL-1 α, IL-1β, IL-6, and IL-17 belong to the members of Th17 cell cytokine family. IL-17A, secreted by Th17 cells, was found upregulated in human inflammatory and autoimmune disease (148). IL-1α −889C/T T allele had a favorable relationship in the prevention of periodontitis risk with OR (95% CI), 1.12 (0.99–1.25) (**Figure 4A**). LFK index showed that there was major asymmetry and publication bias for the result of IL-1α −889C/T T allele model (**Figure 4B**). In contrast, IL-1α −889C/T C allele also had a strong relationship with the periodontitis development with OR (95% CI), 0.75 (0.66–0.85) (**Figure 4C**). LFK index indicated that there was major asymmetry and publication bias for the result of IL-1α −889C/T C allele model (**Figure 4D**).

IL-1β −511C>T polymorphism had a very strong relationship in the periodontitis risk with OR (95% CI), 0.50 (0.30–0.71) (**Figure 4E**). LFK index showed that there was major asymmetry and publication bias for the result of IL-1β −511C>T polymorphism (**Figure 4F**). Similarly, IL-1β 3954C>T polymorphism also had a very strong relationship with the periodontitis development with OR (95% CI), 0.66 (0.52–0.80) (**Figure 4G**). LFK index indicated that there was major asymmetry and publication bias for the result of IL-1β 3954C>T polymorphism (**Figure 4H**).

IL-6 −174G/C G allele model had a weak relationship in the prevention of periodontitis risk with OR (95% CI), 1.10 (0.82–1.38) (**Figure 4I**). LFK index showed that there was major asymmetry and publication bias for the result of IL-6 −174G/C G allele model (**Figure 4J**). Similarly, IL-6 −174G/C C allele model also had a weak relationship in the prevention of periodontitis development with OR (95% CI), 1.09 (0.81–1.36) (**Figure 4K**). LFK index indicated that there was major asymmetry and publication bias for the result of IL-6 −174G/C C allele model (**Figure 4L**).

IL-17 rs2275913 polymorphism had a weak relationship in the periodontitis risk with OR (95% CI), 0.92 (0.45–1.39) (**Figure 4M**). LFK index showed that there was no asymmetry or publication bias for the result of IL-17 rs2275913 polymorphism (**Figure 4N**). Taken together, in the members of Th17 cytokine family, different IL-1 α polymorphisms may have inverse functions in the pathogenesis of periodontitis. IL-1β is a significant cytokine biomarker in the periodontitis development. IL-6 may have protective function in the inflammatory responses of periodontitis, and IL-17 has a weak relationship with the inflammatory responses.

### Treg Cell Cytokines

**Figure 5:**
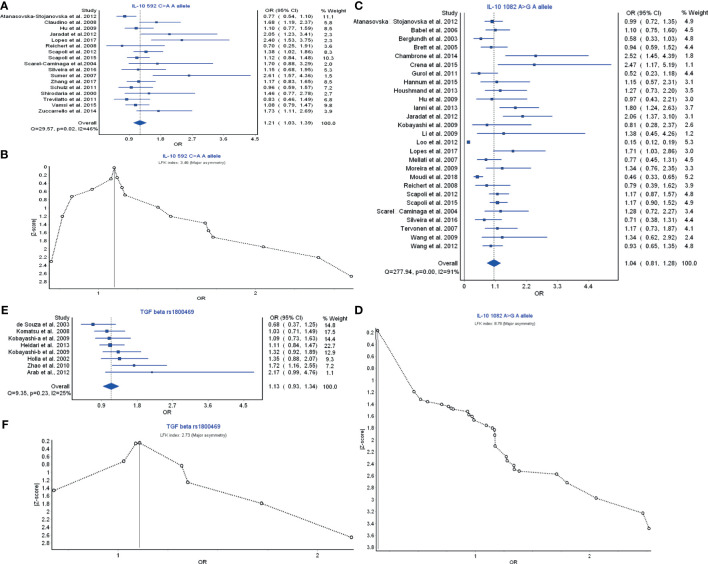
The association of Treg-related cytokine polymorphism with chronic periodontitis. **(A)** Forest plot of the association of IL-10 592C>A A allele polymorphism with chronic periodontitis. **(B)** The Doi plot is used to visualize the asymmetry of the association of IL-10 592C>A A allele polymorphism with chronic periodontitis using the LFK index. LFK index zero represents complete symmetry, the limits of symmetry were set at ±1, and values beyond ±1 were inconsistent with symmetry. **(C)** Forest plot of the association of IL-10 1082A>G A allele polymorphism with chronic periodontitis. **(D)** The Doi plot is used to visualize the asymmetry of the association of IL-10 1082A>G A allele polymorphism with chronic periodontitis using the LFK index. **(E)** Forest plot of the association of TGF-β rs1800469 polymorphism with chronic periodontitis. **(F)** The Doi plot is used to visualize the asymmetry of the association of TGF-β rs1800469 polymorphism with chronic periodontitis using the LFK index. LFK index zero represents complete symmetry, the limits of symmetry were set at ±1, and values beyond ±1 were inconsistent with symmetry.

Treg cell cytokines include TGF-β; and the IL-10 family, and TGF-β and IL-10 are the most relevant members for immune regulation. IL-10 592C>A A allele model had a strong relationship in the prevention of periodontitis risk with OR (95% CI), 1.21 (1.03–1.39) (**Figure 5A**). LFK index showed that there was major asymmetry and publication bias for the result of IL-10 592C>A A allele model (**Figure 5B**). IL-10 1082A>G A allele model also had a weak relationship with the periodontitis development with OR (95% CI), 1.04 (0.81–1.28) (**Figure 5C**). LFK index indicated that there was major asymmetry and publication bias for the result of L-10 1082A>G A allele model (**Figure 5D**). TGF-β rs1800469 polymorphism had a relationship in the prevention of periodontitis risk with OR (95% CI), 1.13 (0.93–1.34) (**Figure 5E**). LFK index showed that there was major asymmetry and publication bias for the result of TGF-β rs1800469 polymorphism (**Figure 5F**). Taken together, the members of Treg cell cytokines may have a protective function in the periodontitis risk by reducing the inflammatory responses.

## Discussion

**Figure 6:**
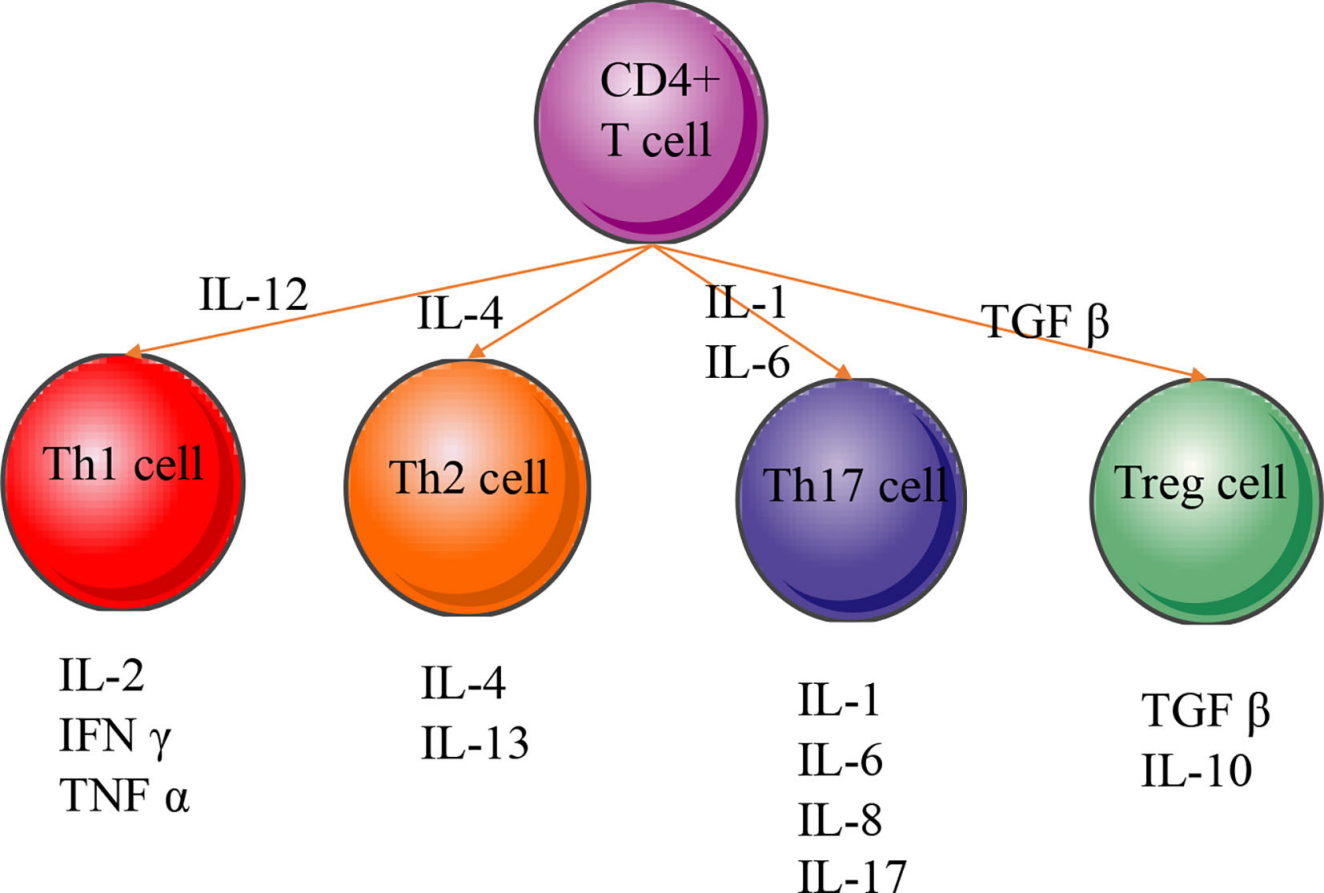
CD4+ T-cell differentiation and cellular phenotype. Naive CD4+ T cells differentiate into many different types of T helper (Th) or regulatory T (Treg) cells. The regulating transcription factors of subsets and their characteristic effector cytokines are indicated in the figure. IL‐1, interleukin‐1; IL‐2, interleukin-2; IL‐4, interleukin‐4; IL‐6, interleukin‐6; IL‐8, interleukin‐8; IL‐10, interleukin-10; IL‐13, interleukin‐13; IL‐17, interleukin‐17; IFN-γ, interferon gamma; TNF-α, tumor necrosis factor alpha; TGF-β, transforming growth factor beta.

Pro-inflammatory CD4+ T helper cells mainly belong to the Th1, Th2, Th17, and Treg cells, which secrete various cytokines and are closely associated with periodontal diseases (**Figure 6**) (149). The present meta-analysis showed that the polymorphisms of the members IL-2 and TNF-α of Th1 cytokine family may be associated with the pathogenesis of periodontitis or the prevention of periodontitis risk, while the polymorphism of IFN-γ is not related to periodontitis risk. The result is consistent with previous meta-analysis that IFN-γ may not contribute to periodontitis susceptibility (150). Cytokine receptors also can induce inflammatory signals by binding specifically to certain types of cytokines. The activity of IL-2 receptor in the periodontitis patients was also found to be higher than that in the control subjects (151). Previous report also could not find any evidence to support that polymorphisms in an IFN-γ receptor‐1 contributes to periodontitis risk either (152).

TNF-α, one of the essential pro-inflammatory cytokines in periodontitis patients, has been regarded as a potential biomarker for diagnosis of a periodontal disease (153). Anti-TNF-α will be a potential way to prevent periodontitis risk (154). Apical periodontitis patients were treated with anti-TNF-α biologic medications and showed faster healing than the controls (155).

The polymorphisms for the members IL-4 and IL-13 of Th2 cytokine family are not found to be associated with the pathogenesis of periodontitis. The statistical differences for IL-4 receptor polymorphism were insignificant among mild, moderate, and severe chronic periodontitis subjects (156). Further work also did not show that the polymorphisms of IL-4 receptor were associated with the risk of severe chronic periodontitis (157).

For the polymorphisms of the members of Th17 cytokine family, different IL-1α polymorphisms may have inverse actions in the pathogenesis of periodontitis. IL-1β is a noteworthy cytokine biomarker in periodontitis development and progression. IL-6 may have a protective function in the inflammatory responses of periodontitis, and IL-17 has a weak relationship the inflammatory responses. The relationship between interleukin-1 receptor and periodontitis risk was seldom reported. The polymorphism (rs2234663) of interleukin-1 receptor antagonist (IL-1ra), an agent that binds to interleukin-1 receptor, was found to contribute to serious periodontitis susceptibility (158). Salivary level of IL-1Ra was also reported to be statistically higher in the periodontitis patients than the controls (159). Gingival crevicular fluid-soluble IL-6 receptor was also higher in the inflammatory sites than in the healthy controls in periodontitis patients (160).

Clinical evidence shows that IL-1β, a pro-inflammatory cytokine, is raised and contributes to periodontitis susceptibility. Evaluated levels of IL-1β initiate a chain of inflammatory responses and increase bone resorption (161). Therefore, IL-1β is an important therapeutic target for periodontitis. The application of IL-1ra (a natural inhibitor of IL-1β) suggests the possible function of IL-1ra in controlling periodontal inflammation in an experimental periodontitis model (162).

The present study showed that IL-2 −330T allele had a strong relationship with the periodontitis risk (**Figure 2C**), which was consistent with the previous report that the expression of IL-2 was increased in the tenacity stage of periodontitis and that IL-2 exerts an inhibitory function in the development of periodontitis (163). IL-2 −330T allele may affect its normal protective function. Preclinical experiments indicated that IL-2/anti-IL-2-antibody complexes activated the percent of Treg cells and reduced the inflammatory responses in various diseases (164, 165).

The present findings showed that the members IL-4 and IL-13 of Th2 cytokine family may not be associated with the pathogenesis of periodontitis. The results are inconsistent with the previous report that IL-4 has been found to be decreased in chronic periodontitis subjects and increased after therapy, which implies that IL-4 in Th2 cells may have protective effects in the prevention of periodontitis (166). Furthermore, it has been also reported that IL-4 can control pro-inflammatory cytokine levels (167) and restrain the occurrence of osteoclastogenesis (168).

The present findings showed that the members of Treg cell cytokines may have protective function in the periodontitis risk by reducing the inflammatory responses, which are consistent with popular reports. TGF-β and IL-10 are the main effector cytokines in Treg cells and may have synergetic functions (169, 170). Treg cells might gather at infecting sites of dental infection and might reduce immune actions and bone resorption in the periodontal area (171). The upregulation of TGF-β results in collagen production and reduces collagen-destroying MMP-1 production, which will be beneficial to protect against periodontitis (172). IL-10 polymorphism is of high clinical relevance and a valuable marker to recognize patients who are at higher risk for periodontitis (173). On the other hand, regulatory T cells play an important role in modulating the immunity in periodontitis patients by defending against inflammation and autoimmunity (174). Modulating the Th17/Treg imbalance during periodontitis may be a promising approach to cure periodontitis (175, 176) by affecting the levels of TGF-β and IL-10 (177).

### Strengths and Limitations

This meta‐analysis has several strengths, including an unrestricted search process and duplicate review procedures for the search and strict assessments of the risk of bias and the quality of literature.

There are some limitations in the present paper. First, although a broad search in different databases of various cytokines was carried out, it is difficult to approve that whether all the studies for exploring the relationship between these genetic polymorphisms of various cytokines and periodontitis risk were included in the present study. Second, there were obvious heterogeneities and potential publication biases in these meta-analyses. To reduce some potential heterogeneities, the related literatures were removed; however, some heterogeneities still existed. Third, we used Doi plot to probe the occurrence of publication bias in the studies used for our meta‐analysis, and the results showed that all LFK indexes showed the major asymmetry due to publication bias. Therefore, to understand whether the different Th cell cytokines were associated with periodontitis, more unbiased data are required. IL-23 plays a critical role in the development of periodontitis (9, 178–181). However, the present research focuses on the relationship between cytokine polymorphisms and periodontitis and not between the cytokine level and periodontitis. We have tried to investigate the relationship between IL-23 polymorphisms and periodontitis. Unfortunately, the related contents were seldom reported, and the meta-analysis was not performed.

## Conclusions

Within the limitations of this study, the present meta‐analysis indicated that genetic polymorphism of Th17 cytokines might be a risk factor for the development of periodontitis, while Treg cells may show protective function in the prevention of periodontitis inflammation. Further studies on larger population will be needed to verify these findings.

## Data Availability Statement

The original contributions presented in the study are included in the article/**Supplementary Material**. Further inquiries can be directed to the corresponding author.

## Author Contributions

XL and HL contributed to conception, design of the study, collect all literatures, analyze all data and wrote the manuscript. All authors contributed to the article and approved the submitted version.

## Funding

The present project was supported by the Department of Science and Technology of Jilin Province (Grant No. 20200201519JC).

## Conflict of Interest

The authors declare that the research was conducted in the absence of any commercial or financial relationships that could be construed as a potential conflict of interest.

## Publisher’s Note

All claims expressed in this article are solely those of the authors and do not necessarily represent those of their affiliated organizations, or those of the publisher, the editors and the reviewers. Any product that may be evaluated in this article, or claim that may be made by its manufacturer, is not guaranteed or endorsed by the publisher.
