# Supplementary material for: A Systematic Review and Meta-Analysis on Multiple Cytokine Gene Polymorphisms in the Pathogenesis of Periodontitis
Source: Front Immunol. 2022 Jan 3;12:713198. doi: 10.3389/fimmu.2021.713198 (PMC8761621; doi:10.3389/fimmu.2021.713198)
Supplement: Supplementary file 1 [file Table_1.docx]

Table S1. Association between - 330 T/G polymorphism in interleukin-2 gene and periodontitis

|  | Cases |  |  | Control |  |  | HWE | references |
| --- | --- | --- | --- | --- | --- | --- | --- | --- |
|  | GG | TG | TT | GG | TG | TT |  |  |
| Majumder, 2019 | 72 | 62 | 23 | 90 | 79 | 31 | 0.013 | ^3^ |
| Li, 2012 | 40 | 14 | 68 | 43 | 111 | 378 | 0.000 | ^4^ |
| Reichert, 2009 | 8 | 15 | 35 | 16 | 51 | 64 | 0.019 | ^5^ |
| Scarel-Caminaga, 2002 | 4 | 29 | 36 | 2 | 16 | 26 | 0.553 | ^6^ |
| Vahabi, 2017 | 0 | 0 | 99 | 0 | 0 | 75 |  | ^7^ |

**References**

1. da Silva FRP, Galeno JG, Leal A, et al. Non-significant association between - 330 T/G polymorphism in interleukin-2 gene and chronic periodontitis: findings from a meta-analysis. *BMC Oral Health*. Feb 19 2020;20(1):58. doi:10.1186/s12903-020-1034-8

2. Gomes MS, Blattner TC, Sant'Ana Filho M, et al. Can apical periodontitis modify systemic levels of inflammatory markers? A systematic review and meta-analysis. *J Endod*. Oct 2013;39(10):1205-17. doi:10.1016/j.joen.2013.06.014

3. Majumder P, Panda SK, Ghosh S, Dey SK. Interleukin gene polymorphisms in chronic periodontitis: A case-control study in the Indian population. *Arch Oral Biol*. May 2019;101:156-164. doi:10.1016/j.archoralbio.2019.03.015

4. Li G, Yue Y, Tian Y, et al. Association of matrix metalloproteinase (MMP)-1, 3, 9, interleukin (IL)-2, 8 and cyclooxygenase (COX)-2 gene polymorphisms with chronic periodontitis in a Chinese population. *Cytokine*. 2012;60(2):552-560.

5. Reichert S, Machulla HK, Klapproth J, et al. Interleukin-2 -330 and 166 gene polymorphisms in relation to aggressive or chronic periodontitis and the presence of periodontopathic bacteria. *J Periodontal Res*. Oct 2009;44(5):628-35. doi:10.1111/j.1600-0765.2008.01173.x

6. Scarel-Caminaga RM, Trevilatto PC, Souza AP, Brito RB, Line SR. Investigation of an IL-2 polymorphism in patients with different levels of chronic periodontitis. *J Clin Periodontol*. Jul 2002;29(7):587-91. doi:10.1034/j.1600-051x.2002.290701.x

7. Vahabi S, Nazemisalman B, Hosseinpour S, Salavitabar S, Aziz A. Interleukin-2, -16, and -17 gene polymorphisms in Iranian patients with chronic periodontitis. *J Investig Clin Dent*. May 2018;9(2):e12319. doi:10.1111/jicd.12319
