# Supplementary material for: A Systematic Review and Meta-Analysis on Multiple Cytokine Gene Polymorphisms in the Pathogenesis of Periodontitis
Source: Front Immunol. 2022 Jan 3;12:713198. doi: 10.3389/fimmu.2021.713198 (PMC8761621; doi:10.3389/fimmu.2021.713198)
Supplement: Supplementary file 2 [file Table_2.docx]

Table s2: the association between IFN‐γ874A/T polymorphism and periodontitis risk

| **Studies and years** | OR (95% CI) | References |
| --- | --- | --- |
| Heidari et al. 2015 | 0.49 (0.28 0.86) | ^3^ |
| lanni et al. 2013 | 1.48 (0.77 2.83) | ^4^ |
| Loo et al. 2012 | 1.11 (0.85, 1.45) | ^5^ |
| Holla et al. 2011 | 1.16 (0.78, 1.73) | ^6^ |
| Erciyas et al. 2010 | 0.91 (0.40.2.03) | ^7^ |
| Reichert et al. 2008 | 0.91 (0.49 1.69) | ^8^ |
| Babel et al. 2006 | 0.85 (0.50.1.44) | ^9^ |

**Dominant model**

| **Studies and years** | OR (95% CI) | References |  |
| --- | --- | --- | --- |
| Heidari et al. 2015 | 0 66 (0.33, 1.34) | ^3^ |  |
| lanni et al. 2013 | 0 71 (0.38, 1.33) | ^4^ |  |
| Loo et al. 2012 | 1.04 (0.83, 1.32) | ^5^ |  |
| Holla et al. 2011 | 1 36 (0.89, 2.07) | ^6^ |  |
| Erciyas et al. 2010 | 2 41 (0.80, 7.25) | ^7^ |  |
| Reichert et al. 2008 | 0.79 (0.39, 1.56) | ^8^ |  |

**Recessive model**

**References**

1. Stadler AF, Angst PD, Arce RM, Gomes SC, Oppermann RV, Susin C. Gingival crevicular fluid levels of cytokines/chemokines in chronic periodontitis: a meta-analysis. *J Clin Periodontol*. Sep 2016;43(9):727-45. doi:10.1111/jcpe.12557

2. Shi Q, Cai C, Xu J, Liu J, Liu H, Huo N. Is there an association between IFN-gamma +874A/T polymorphism and periodontitis susceptibility?: A meta-analysis. *Medicine (Baltimore)*. Jun 2017;96(25):e7288. doi:10.1097/MD.0000000000007288

3. Heidari Z, Mahmoudzadeh-Sagheb H, Hashemi M, Ansarimoghaddam S, Moudi B, Sheibak N. Association between IFN-c? 874 A/T and IFN-cR1 (-611A/G,? 189T/G and? 95C/T) gene polymorphisms and chronic periodontitis in a sample of Iranian population. *Int J Dent*. 2015;

4. Ianni M, Bruzzesi G, Pugliese D, et al. Variations in inflammatory genes are associated with periodontitis. *Immun Ageing*. Oct 1 2013;10(1):39. doi:10.1186/1742-4933-10-39

5. Loo WT, Fan CB, Bai LJ, et al. Gene polymorphism and protein of human pro- and anti-inflammatory cytokines in Chinese healthy subjects and chronic periodontitis patients. *J Transl Med*. Sep 19 2012;10 Suppl 1:S8. doi:10.1186/1479-5876-10-S1-S8

6. Holla LI, Hrdlickova B, Linhartova P, Fassmann A. Interferon-γ+ 874A/T polymorphism in relation to generalized chronic periodontitis and the presence of periodontopathic bacteria. *archives of oral biology*. 2011;56(2):153-158.

7. Erciyas K, Pehlivan S, Sever T, Igci M, Arslan A, Orbak R. Association between TNF-alpha, TGF-beta1, IL-10, IL-6 and IFN-gamma gene polymorphisms and generalized aggressive periodontitis. *Clin Invest Med*. Apr 1 2010;33(2):E85. doi:10.25011/cim.v33i2.12346

8. Reichert S, Machulla HK, Klapproth J, et al. Interferon‐gamma and interleukin‐12 gene polymorphisms and their relation to aggressive and chronic periodontitis and key periodontal pathogens. *Journal of periodontology*. 2008;79(8):1434-1443.

9. Babel N, Cherepnev G, Babel D, et al. Analysis of tumor necrosis factor-alpha, transforming growth factor-beta, interleukin-10, IL-6, and interferon-gamma gene polymorphisms in patients with chronic periodontitis. *J Periodontol*. Dec 2006;77(12):1978-83. doi:10.1902/jop.2006.050315
