# Supplementary material for: A Systematic Review and Meta-Analysis on Multiple Cytokine Gene Polymorphisms in the Pathogenesis of Periodontitis
Source: Front Immunol. 2022 Jan 3;12:713198. doi: 10.3389/fimmu.2021.713198 (PMC8761621; doi:10.3389/fimmu.2021.713198)
Supplement: Supplementary file 3 [file Table_3.docx]

Table S3, association between TNF-α308G/A polymorphism and periodontitis

| Studies and years | OR | (lower limit | Upper limit) |  | |  |
| --- | --- | --- | --- | --- | --- | --- |
| Brett et al. 2005 | 0.78 | (0.39, | 1.56) | ^9^ | |  |
| de Sa et al. 2007 | 2.76 | (1.22, | 6.26) | ^8^ | |  |
| Dominguez-Perez et al. 2017 | 1 | (0.42, | 2.38) | ^10^ | |  |
| Ebadian et al. 2013 | 1.56 | (0.90, | 2.72) | ^11^ | |  |
| Endo et al. 2001 | 0.13（ | 0.2， | 6.30） | ^12^ | |  |
| Fassmann et al. 2003 | 0.83 | (0.45, | 1.52) | ^13^ | |  |
| Folwaczny et al. 2004 | 1.47 | (0.75, | 2.87) | ^14^ | |  |
| Guzeldemir et al. 2008 | 2.73 | 1.04 | 7.21 | ^15^ | |  |
| lanni et al. 2013 | 0.39 | (0.18, | 0.89) | ^16^ | |  |
| Lin et al. 2012 | 0.85 | (0.43, | 1.72) | ^17^ | |  |
| Liu et al. 2011 | 3.04 | (1.09, | 8.51) | ^18^ | |  |
| Ma et al. 2011 | 2.37 | (1.32, | 4.24) | ^19^ | |  |
| Majumder et al. 2018 | 2.98 | (1.90, | 4.70) | ^20^ | |  |
| Moreira et al. 2009 | 1.66 | (0.64, | 4.31) | ^21^ | |  |
| Ozer Yucel et al. 2015 | 2.68 | (0.90, | 8.00) | ^22^ | |  |
| Pang et al. 2005 | 1.54 | (0.71, | 3.34) | ^23^ | |  |
| Sakellari et al. 2006 | 0.53 | (0.22, | 1.24) | ^24^ | |  |
| Schulz et al. 2014 | 1.22 | (0.59, | 2.50) | ^25^ | |  |
| Sharma et al. 2014 | 9.3 | (1.12, | 77.38) | ^26^ | |  |
| Trombone et al. 2009 | 1.54 | (0.90, | 2.64) | ^27^ | |  |
| Yang et al. 2013 | 1.05 | (0.68, | 1.62) | ^28^ | |  |
| Zhong et al. 2005 | 1.23 | (0.60, | 2.52) | ^29^ | |  |
|  |  |  |  |  |  | |

1. Jakovljevic A, Nikolic N, Jacimovic J, et al. TNF-alpha -308 G/A single nucleotide polymorphism and apical periodontitis: an updated systematic review and meta-analysis. *J Endod*. Mar 25 2021;doi:10.1016/j.joen.2021.03.007

2. Li Y, Yang J, Wu X, Sun W. TNF-alpha polymorphisms might influence predisposition to periodontitis: A meta-analysis. *Microb Pathog*. Jun 2020;143:104113. doi:10.1016/j.micpath.2020.104113

3. Salles AG, Antunes LAA, Carvalho PA, Kuchler EC, Antunes LS. Association Between Apical Periodontitis and TNF-alpha -308 G>A Gene Polymorphism: A Systematic Review and Meta-Analysis. *Braz Dent J*. Sep-Oct 2017;28(5):535-542. doi:10.1590/0103-6440201701491

4. Shi LX, Zhang L, Zhang DL, et al. Association between TNF-alpha G-308A (rs1800629) polymorphism and susceptibility to chronic periodontitis and type 2 diabetes mellitus: A meta-analysis. *J Periodontal Res*. Apr 2021;56(2):226-235. doi:10.1111/jre.12820

5. Wei XM, Chen YJ, Wu L, Cui LJ, Hu DW, Zeng XT. Tumor necrosis factor-alpha G-308A (rs1800629) polymorphism and aggressive periodontitis susceptibility: a meta-analysis of 16 case-control studies. *Sci Rep*. Jan 11 2016;6:19099. doi:10.1038/srep19099

6. Xu L, Liu C, Zheng Y, et al. Association of TNF-alpha-308G/A, -238G/A, -863C/A, -1031T/C, -857C/T polymorphisms with periodontitis susceptibility: Evidence from a meta-analysis of 52 studies. *Medicine (Baltimore)*. Sep 4 2020;99(36):e21851. doi:10.1097/MD.0000000000021851

7. Amaya M, Criado L, Blanco B, et al. Polymorphisms of pro‐inflammatory cytokine genes and the risk for acute suppurative or chronic nonsuppurative apical periodontitis in a C olombian population. *International endodontic journal*. 2013;46(1):71-78.

8. de Sa AR, Moreira PR, Xavier GM, et al. Association of CD14, IL1B, IL6, IL10 and TNFA functional gene polymorphisms with symptomatic dental abscesses. *Int Endod J*. Jul 2007;40(7):563-72. doi:10.1111/j.1365-2591.2007.01272.x

9. Brett P, Zygogianni P, Griffiths G, et al. Functional gene polymorphisms in aggressive and chronic periodontitis. *Journal of dental research*. 2005;84(12):1149-1153.

10. Domínguez-Pérez RA, Loyola-Rodriguez JP, Abud-Mendoza C, Alpuche-Solis AG, Ayala-Herrera JL, Martínez-Martínez RE. Association of cytokines polymorphisms with chronic peridontitis and rheumatoid arthritis in a Mexican population. *Acta Odontologica Scandinavica*. 2017;75(4):243-248.

11. Ebadian AR, Radvar M, Afshari JT, et al. Gene polymorphisms of TNF-α and IL-1β are not associated with generalized aggressive periodontitis in an Iranian subpopulation. *Iranian Journal of Allergy, Asthma and Immunology*. 2013:345-351.

12. Endo M, Tai H, Tabeta K, Kobayashi T, Yamazaki K, Yoshie H. Analysis of single nucleotide polymorphisms in the 5'-flanking region of tumor necrosis factor-alpha gene in Japanese patients with early-onset periodontitis. *J Periodontol*. Nov 2001;72(11):1554-9. doi:10.1902/jop.2001.72.11.1554

13. Fassmann A, Holla LI, Buckova D, Vasku A, Znojil V, Vanek J. Polymorphisms in the +252(A/G) lymphotoxin-alpha and the -308(A/G) tumor necrosis factor-alpha genes and susceptibility to chronic periodontitis in a Czech population. *J Periodontal Res*. Aug 2003;38(4):394-9. doi:10.1034/j.1600-0765.2003.00661.x

14. Folwaczny M, Glas J, Török HP, Mende M, Folwaczny C. Lack of association between the TNFα G− 308 A promoter polymorphism and periodontal disease. *Journal of clinical periodontology*. 2004;31(6):449-453.

15. Guzeldemir E, Gunhan M, Ozcelik O, Tastan H. Interleukin-1 and tumor necrosis factor-alpha gene polymorphisms in Turkish patients with localized aggressive periodontitis. *J Oral Sci*. Jun 2008;50(2):151-9. doi:10.2334/josnusd.50.151

16. Ianni M, Bruzzesi G, Pugliese D, et al. Variations in inflammatory genes are associated with periodontitis. *Immun Ageing*. Oct 1 2013;10(1):39. doi:10.1186/1742-4933-10-39

17. Lin XH, Chen L, Wu B, Wei B. Association of the tumour necrosis factor-α 308 gene polymorphism and susceptibility to severe chronic periodontitis. *Chinese Journal of Birth Health & Heredity*. 2012;20(06):13-14.

18. Bo L, Ning Y, Li-si T, Jing-bo L, Yan G, YA-ping P. A study of frequency of TNF alpha gene with type 2 diabetes mellitus with chronic periodontitis. *Shanghai Journal of Stomatology*. 2011;20(2)

19. Ma M, Li G, Han C, Huang Y. Correlation study on polymorphisms of the Interleukin-1 and tumor necrosis factoralpha gene in Hui patients with chronic periodontitis in Ningxia. *Journal of Modern Stomatology*. 2011;25:94-97.

20. Majumder P, Thou K, Bhattacharya M, Nair V, Ghosh S, Dey SK. Association of tumor necrosis factor-alpha (TNF-alpha) gene promoter polymorphisms with aggressive and chronic periodontitis in the eastern Indian population. *Biosci Rep*. Aug 31 2018;38(4)doi:10.1042/BSR20171212

21. Moreira P, Costa J, Gomez R, Gollob K, Dutra W. TNFA and IL10 gene polymorphisms are not associated with periodontitis in Brazilians. *The Open Dentistry Journal*. 2009;3:184.

22. Ozer Yucel O, Berker E, Mesci L, Eratalay K, Tepe E, Tezcan I. Analysis of TNF-alpha (-308) polymorphism and gingival crevicular fluid TNF-alpha levels in aggressive and chronic periodontitis: A preliminary report. *Cytokine*. Apr 2015;72(2):173-7. doi:10.1016/j.cyto.2015.01.001

23. Pang R, Chen K, Zhang J, Xu C, Zhang X. Association of TNFA-308 gene polymorphisms with susceptibility to chronic periodontitis in Chinese patients. *Shanghai kou qiang yi xue= Shanghai journal of stomatology*. 2005;14(6):586-589.

24. Sakellari D, Katsares V, Georgiadou M, Kouvatsi A, Arsenakis M, Konstantinidis A. No correlation of five gene polymorphisms with periodontal conditions in a Greek population. *Journal of clinical periodontology*. 2006;33(11):765-770.

25. Schulz S, Reichert S, Streetz K, et al. Tumor necrosis factor-alpha and oral inflammation in patients with Crohn disease. *J Periodontol*. Oct 2014;85(10):1424-31. doi:10.1902/jop.2014.130644

26. Sharma N, Joseph R, Arun R, Chandni R, Srinivas KL, Banerjee M. Cytokine gene polymorphism (interleukin-1beta +3954, Interleukin-6 [-597/-174] and tumor necrosis factor-alpha -308) in chronic periodontitis with and without type 2 diabetes mellitus. *Indian J Dent Res*. May-Jun 2014;25(3):375-80. doi:10.4103/0970-9290.138343

27. Trombone A, Cardoso C, Repeke C, et al. Tumor necrosis factor‐alpha− 308G/A single nucleotide polymorphism and red‐complex periodontopathogens are independently associated with increased levels of tumor necrosis factor‐α in diseased periodontal tissues. *Journal of periodontal research*. 2009;44(5):598-608.

28. Yang W, Jia Y, Wu H. Four tumor necrosis factor alpha genes polymorphisms and periodontitis risk in a Chinese population. *Hum Immunol*. Dec 2013;74(12):1684-7. doi:10.1016/j.humimm.2013.08.009

29. Zhong L, Zhang Y, Liu Y-s, Nie J, Wang X. Association between tumour necrosis factor A-308 genotype and chronic periodontitis of Uighur patients. *Chinese Journal of Conservative Dentistry*. 2005;15:550-552.
