# Supplementary material for: A Systematic Review and Meta-Analysis on Multiple Cytokine Gene Polymorphisms in the Pathogenesis of Periodontitis
Source: Front Immunol. 2022 Jan 3;12:713198. doi: 10.3389/fimmu.2021.713198 (PMC8761621; doi:10.3389/fimmu.2021.713198)
Supplement: Supplementary file 4 [file Table_4.docx]

Table S4. the association between IL-4 -590C/T polymorphism and susceptibility to periodontitis, T versus C allele comparison in all study participants.

|  | cases | | controls | | |  |  |  |  | |
| --- | --- | --- | --- | --- | --- | --- | --- | --- | --- | --- |
| Studies and years | events | total | | events | total | OR | 95% CI | References | |  |
| Anovazzi et al. 2010 | 107 | 250 | | 78 | 250 | 1.65 | 1.14; 2.38 | ^1^ | |  |
| Atanasovska-Stojanovska et al. 2011 | 54 | 184 | | 195 | 572 | 0.8 | 0.56; 1.15 | ^2^ | |  |
| Byung et al. 2003 | 46 | 64 | | 225 | 284 | 0.67 | 0.36; 1.24 | ^3^ | |  |
| Chen et al. 2012 | 289 | 396 | | 195 | 256 | 0.84 | 0.59; 1.21 | ^4^ | |  |
| Gonzales et al. 2004 | 28 | 60 | | 18 | 46 | 1.36 | 0.62; 2.97 | ^5^ | |  |
| Gonzales et al. 2004 | 21 | 62 | | 13 | 60 | 1.85 | 0.82; 4.16 | ^5^ | |  |
| Gonzales et al. 2007 | 44 | 116 | | 34 | 102 | 1.22 | 0.70; 2.13 | ^6^ | |  |
| Holla et al. 2008 | 70 | 388 | | 44 | 316 | 1.36 | 0.90; 2.05 | ^7^ | |  |
| Hooshmand et al. 2008a | 10 | 54 | | 34 | 112 | 0.52 | 0.24; 1.16 | ^8^ | |  |
| Hooshmand et al. 2008b | 11 | 52 | | 34 | 112 | 0.62 | 0.28; 1.34 |  | |  |
| Kara et al. 2007 | 30 | 150 | | 25 | 146 | 1.21 | 0.67; 2.18 | ^9^ | |  |
| Loo et al. 2012 | 673 | 880 | | 773 | 1700 | 3.9 | 3.25; 4.68 | ^10^ | |  |
| Scarel-Caminaga et al. 2003 | 34 | 100 | | 23 | 74 | 1.14 | 0.60; 2.17 | ^11^ | |  |

References

1. Anovazzi G, Kim YJ, Viana AC, et al. Polymorphisms and haplotypes in the interleukin-4 gene are associated with chronic periodontitis in a Brazilian population. *J Periodontol*. Mar 2010;81(3):392-402. doi:10.1902/jop.2009.090392

2. Atanasovska-Stojanovska A, Trajkov D, Nares S, Angelov N, Spiroski M. IL4 gene polymorphisms and their relation to periodontal disease in a Macedonian population. *Human Immunology*. 2011;72(5):446-450.

3. Kang BY, Choi YK, Choi WH, et al. Two polymorphisms of interleukin-4 gene in Korean adult periodontitis. *Arch Pharm Res*. Jun 2003;26(6):482-6. doi:10.1007/BF02976867

4. Chen D, Wei N, Bao X, et al. Analysis of correlation between IL-6, IL-6R and IL-4 single nucleotide polymorphism and the susceptibility of chronic periodontitis among Shanghai patients of Han nationality. *Stomatology*. 2012;32:518-520.

5. Gonzales JR, Kobayashi T, Michel J, Mann M, Yoshie H, Meyle J. Interleukin-4 gene polymorphisms in Japanese and Caucasian patients with aggressive periodontitis. *J Clin Periodontol*. May 2004;31(5):384-9. doi:10.1111/j.1600-051X.2004.00492.x

6. Gonzales JR, Mann M, Stelzig J, Bodeker RH, Meyle J. Single-nucleotide polymorphisms in the IL-4 and IL-13 promoter region in aggressive periodontitis. *J Clin Periodontol*. Jun 2007;34(6):473-9. doi:10.1111/j.1600-051X.2007.01086.x

7. Holla LI, Fassmann A, Augustin P, Halabala T, Znojil V, Vanek J. The association of interleukin-4 haplotypes with chronic periodontitis in a Czech population. *J Periodontol*. Oct 2008;79(10):1927-33. doi:10.1902/jop.2008.080035

8. Hooshmand B, Hajilooi M, Rafiei A, Mani-Kashani KH, Ghasemi R. Interleukin-4 (C-590T) and interferon-gamma (G5644A) gene polymorphisms in patients with periodontitis. *J Periodontal Res*. Feb 2008;43(1):111-5. doi:10.1111/j.1600-0765.2007.01006.x

9. Kara N, Keles GC, Sumer P, et al. Association of the polymorphisms in promoter and intron regions of the interleukin-4 gene with chronic periodontitis in a Turkish population. *Acta Odontol Scand*. Oct 2007;65(5):292-7. doi:10.1080/00016350701644040

10. Loo WT, Fan CB, Bai LJ, et al. Gene polymorphism and protein of human pro- and anti-inflammatory cytokines in Chinese healthy subjects and chronic periodontitis patients. *J Transl Med*. Sep 19 2012;10 Suppl 1:S8. doi:10.1186/1479-5876-10-S1-S8

11. Scarel-Caminaga RM, Trevilatto PC, Souza AP, Brito RB, Jr., Line SR. Investigation of IL4 gene polymorphism in individuals with different levels of chronic periodontitis in a Brazilian population. *J Clin Periodontol*. Apr 2003;30(4):341-5. doi:10.1034/j.1600-051x.2003.00317.x

12. Chen D, Zhang T-l, Wang X. Association between polymorphisms in interleukins 4 and 13 genes and chronic periodontitis in a Han Chinese population. *BioMed research international*. 2016;2016

13. Jain N, Joseph R, Balan S, Arun R, Banerjee M. Association of interleukin-4 and interleukin-17F polymorphisms in periodontitis in Dravidian ethnicity. *Indian J Hum Genet*. Jan 2013;19(1):58-64. doi:10.4103/0971-6866.112891
