# Supplementary material for: A Systematic Review and Meta-Analysis on Multiple Cytokine Gene Polymorphisms in the Pathogenesis of Periodontitis
Source: Front Immunol. 2022 Jan 3;12:713198. doi: 10.3389/fimmu.2021.713198 (PMC8761621; doi:10.3389/fimmu.2021.713198)
Supplement: Supplementary file 5 [file Table_5.docx]

Table S5. The association between IL-13 -1112C/T polymorphisms and periodontitis risk.

| Authors and years | Distribution area | Cases (*n*) | Controls (*n*) | Case genotype | | | Control genotype | | | HWE (*P*) | References |
| --- | --- | --- | --- | --- | --- | --- | --- | --- | --- | --- | --- |
|  |  |  |  | CC | CT | TT | CC | CT | TT |  |  |
| Gonzales et al. 2007 | Germany | 58 | 51 | 19 | 29 | 10 | 17 | 29 | 5 | 0.145 | ^2^ |
| Wu YM1 et al. 2010 | China | 60 | 95 | 47 | 13 | 0 | 52 | 41 | 2 | 0.059 | ^3^ |
| Wu YM2 et al. 2010 | China | 204 | 95 | 117 | 84 | 3 | 52 | 41 | 2 | 0.059 | ^3^ |
| Chen et al. 2013 | China | 278 | 324 | 171 | 67 | 40 | 208 | 92 | 24 | 0.004 | ^4^ |

References

1. Zhang W, Xu P, Chen Z, Cheng Y, Li X, Mao Q. IL-13 -1112 polymorphism and periodontitis susceptibility: a meta-analysis. *BMC Oral Health*. Feb 7 2018;18(1):21. doi:10.1186/s12903-018-0481-y

2. Gonzales JR, Mann M, Stelzig J, Bodeker RH, Meyle J. Single-nucleotide polymorphisms in the IL-4 and IL-13 promoter region in aggressive periodontitis. *J Clin Periodontol*. Jun 2007;34(6):473-9. doi:10.1111/j.1600-051X.2007.01086.x

3. Wu YM, Chuang HL, Ho YP, Ho KY, Tsai CC. Investigation of interleukin‐13 gene polymorphisms in individuals with chronic and generalized aggressive periodontitis in a Taiwanese (Chinese) population. *Journal of periodontal research*. 2010;45(5):695-701.

4. Chen L, Shen Y, Liu L, Li X, Wang T, Wen F. Interleukin-13 -1112 C/T promoter polymorphism confers risk for COPD: a meta-analysis. *PLoS One*. 2013;8(7):e68222. doi:10.1371/journal.pone.0068222
