# Supplementary material for: A Systematic Review and Meta-Analysis on Multiple Cytokine Gene Polymorphisms in the Pathogenesis of Periodontitis
Source: Front Immunol. 2022 Jan 3;12:713198. doi: 10.3389/fimmu.2021.713198 (PMC8761621; doi:10.3389/fimmu.2021.713198)
Supplement: Supplementary file 6 [file Table_6.docx]

Table S6. The association of IL-1alpha -889C/T polymorphism with chronic periodontitis

|  |  |  |  | Experiment | |  | Control |  |  |  | |  | |  |
| --- | --- | --- | --- | --- | --- | --- | --- | --- | --- | --- | --- | --- | --- | --- |
|  | Ethnicity | Case/control |  | events | | total | events | total | OR [95% CI] | References | |  |  |  |
| Al-Hebshi et al. 2012 | Caucasian | 40/40 | 44 | | 80 | 31 | 80 | 1.93 [1.03, 3.63) | | ^5^ |  |  |  |  |
| Armingohar et al. 2014 | Caucasian | 38/36 | 21 | | 76 | 26 | 72 | 0.68 [0.34, 1.35) | | ^6^ |  |  |  |  |
| Boukorrt et al. 2015 | Caucasian | 91/128 | 65 | | 182 | 92 | 256 | 0.99 (0.67, 1.471 | | ^7^ |  |  |  |  |
| Braosi et al. 2012 | Other | 130/116 | 73 | | 260 | 63 | 232 | 1.05 [0.70, 1.56] | | ^8^ |  |  |  |  |
| Brett et al. 2005 | Caucasian | 57/100 | 56 | | 114 | 75 | 200 | 1.61 (1.01, 2.56) | | ^9^ |  |  |  |  |
| Gore et al. 1998 | Caucasian | 32/32 | 17 | | 64 | 16 | 64 | 1.09 [0.49, 2.40) | | ^10^ |  |  |  |  |
| Karasneh et al. 2011 | Caucasian | 100/80 | 81 | | 200 | 54 | 160 | 1.34 [0.87, 2.06) | | ^11^ |  |  |  |  |
| Lamm et al. 2001 | Caucasian | 105/53 | 79 | | 210 | 40 | 106 | 1.00 [0.61, 1.61) | | ^12^ |  |  |  |  |
| Lopez et al. 2005 | Caucasian | 330/101 | 148 | | 660 | 38 | 202 | 1.25 (0.84, 1.86) | | ^13^ |  |  |  |  |
| Lopez et al. 2009 | Caucasian | 224/208 | 143 | | 448 | 98 | 416 | 1.5211.13, 2.06] | | ^14^ |  |  |  |  |
| Puri et al. 2015 | Asian | 20/20 | 24 | | 40 | 16 | 40 | 2.25 (0.92, 5.50) | | ^15^ |  |  |  |  |
| Rogers et al. 2002 | Caucasian | 105/60 | 55 | | 210 | 30 | 120 | 1.06 [0.64, 1.78) | | ^16^ |  |  |  |  |
| Schulz et al. 2011 | Caucasian | 72/89 | 41 | | 144 | 52 | 178 | 0.96 [0.59, 1.57) | | ^17^ |  |  |  |  |
| Shirodaria et al. 2000 | Caucasian | 83/27 | 70 | | 166 | 18 | 54 | 1.46 [0.77, 2.78) | | ^18^ |  |  |  |  |
| Trevilatto et al. 2011 | Other | 69/44 | 37 | | 138 | 27 | 88 | 0.83 [0.46, 1.49) | | ^19^ |  |  |  |  |
| Vamsi et al. 2015 | Asian | 200/200 | 113 | | 400 | 107 | 400 | 1.08 [0.79, 1.47) | | ^20^ |  |  |  |  |
| Zuccarrello et al. 2014 | Caucasian | 101/105 | 66 | | 202 | 46 | 210 | 1.73 [1.11, 2.69) | | ^21^ |  |  |  |  |

**T allele**

|  |  |  |  | Experim |  | Control |  |  |  |  |
| --- | --- | --- | --- | --- | --- | --- | --- | --- | --- | --- |
|  |  |  |  | events | total |  |  |  |  |  |
| Al-Hebshi et al. | 2012 | Caucasian | 40/40 | 36 | 80 | 49 | 80 | 0.52 [0.28, 0.97) | |  |
| Armingohar et a/. | 2014 | Caucasian | 36/38 | 55 | 76 | 46 | 72 | 1.48 [0.74, 2.97) | |  |
| Boukorrt et al. (11) | 2015 | Caucasian | 91/128 | 117 | 182 | 164 | 256 | 1.01 (0.68, 1.50) | |  |
| Braosi et a/. | 2012 | Other | 130/116 | 187 | 260 | 169 | 232 | 0.95 [0.64, 1.42) | |  |
| Brett et al. | 2005 | Caucasian | 57/100 | 58 | 114 | 125 | 200 | 0.62 [0.39, 0.99) | |  |
| Gore et al. | 1998 | Caucasian | 32/32 | 47 | 64 | 48 | 64 | 0.92 [0.42, 2.04) | |  |
| Karasneh et a/. | 2011 | Caucasian | 100/80 | 119 | 200 | 106 | 160 | 0.75 [0.49, 1.15) | |  |
| Lamm et al. | 2001 | Caucasian | 105/53 | 131 | 210 | 66 | 106 | 1.00 (0.62, 1.63) | |  |
| Lopez et al. | 2005 | Caucasian | 330/101 | 512 | 660 | 164 | 202 | 0.80 (0.54, 1.19) | |  |
| Lopez et al. | 2009 | Caucasian | 224/208 | 305 | 448 | 318 | 416 | 0.66 [0.49, 0.89) | |  |
| Puri et al. | 2015 | Asian | 20/20 | 16 | 40 | 24 | 40 | 0.44 [0.18, 1.09) | |  |
| Rogers et al. | 2002 | Caucasian | 105/60 | 155 | 210 | 90 | 120 | 0.94 [0.56, 1.57) | |  |
| Schulz et al. | 2011 | Caucasian | 72/89 | 103 | 144 | 126 | 178 | 1.04 [0.64, 1.68) | |  |
| Shirodaria et al. | 2000 | Caucasian | 83/27 | 96 | 166 | 36 | 54 | 0.69 (0.36, 1.31) | |  |
| Trevilatto et al. | 2011 | Other | 69/44 | 101 | 138 | 61 | 88 | 1.21 (0.67, 2.18) | |  |
| Vamsi et al. | 2015 | Asian | 200/200 | 287 | 400 | 293 | 400 | 0.93 (0.68, 1.27) | |  |
| Zuccarrello et al. | 2014 | Caucasian | 101/105 | 136 | 202 | 164 | 210 | 0.58 [0.37, 0.90) | |  |

**C allele**

**References**

1. da Silva FR, Guimaraes-Vasconcelos AC, de-Carvalho-Franca LF, et al. Relationship between -889 C/T polymorphism in interleukin-1A gene and risk of chronic periodontitis: Evidence from a meta-analysis with new published findings. *Med Oral Patol Oral Cir Bucal*. Jan 1 2017;22(1):e7-e14. doi:10.4317/medoral.21233

2. Feng X, Liu J. Association between IL-1A (-889C/T) polymorphism and susceptibility of chronic periodontitis: A meta-analysis. *Gene*. Mar 1 2020;729:144227. doi:10.1016/j.gene.2019.144227

3. Mao M, Zeng XT, Ma T, He W, Zhang C, Zhou J. Interleukin-1alpha -899 (+4845) C-->T polymorphism increases the risk of chronic periodontitis: evidence from a meta-analysis of 23 case-control studies. *Gene*. Dec 10 2013;532(1):114-9. doi:10.1016/j.gene.2013.09.043

4. Yin WT, Pan YP, Lin L. Association between IL-1alpha rs17561 and IL-1beta rs1143634 polymorphisms and periodontitis: a meta-analysis. *Genet Mol Res*. Feb 5 2016;15(1)doi:10.4238/gmr.15017325

5. Al-Hebshi NN, Shamsan A-aA, Al-Ak'hali MS. Interleukin-1 two-locus haplotype is strongly associated with severe chronic periodontitis among Yemenis. *Molecular biology international*. 2012;2012

6. Armingohar Z, Jorgensen JJ, Kristoffersen AK, Schenck K, Dembic Z. Polymorphisms in the interleukin-1 gene locus and chronic periodontitis in patients with atherosclerotic and aortic aneurysmal vascular diseases. *Scand J Immunol*. May 2014;79(5):338-45. doi:10.1111/sji.12166

7. Boukortt KN, Saidi-Ouahrani N, Boukerzaza B, et al. Association analysis of the IL-1 gene cluster polymorphisms with aggressive and chronic periodontitis in the Algerian population. *Arch Oral Biol*. Oct 2015;60(10):1463-70. doi:10.1016/j.archoralbio.2015.06.018

8. Braosi AP, de Souza CM, Luczyszyn SM, et al. Analysis of IL1 gene polymorphisms and transcript levels in periodontal and chronic kidney disease. *Cytokine*. Oct 2012;60(1):76-82. doi:10.1016/j.cyto.2012.06.006

9. Brett PM, Zygogianni P, Griffiths GS, et al. Functional gene polymorphisms in aggressive and chronic periodontitis. *J Dent Res*. Dec 2005;84(12):1149-53. doi:10.1177/154405910508401211

10. Gore EA, Sanders JJ, Pandey JP, Palesch Y, Galbraith GM. Interleukin-1beta+3953 allele 2: association with disease status in adult periodontitis. *J Clin Periodontol*. Oct 1998;25(10):781-5. doi:10.1111/j.1600-051x.1998.tb02370.x

11. Karasneh JA, Ababneh KT, Taha AH, Al-Abbadi MS, Ollier WE. Investigation of the interleukin-1 gene cluster polymorphisms in Jordanian patients with chronic and aggressive periodontitis. *Arch Oral Biol*. Mar 2011;56(3):269-76. doi:10.1016/j.archoralbio.2010.10.001

12. Laine ML, Farre MA, Gonzalez G, et al. Polymorphisms of the interleukin-1 gene family, oral microbial pathogens, and smoking in adult periodontitis. *J Dent Res*. Aug 2001;80(8):1695-9. doi:10.1177/00220345010800080301

13. Lopez NJ, Jara L, Valenzuela CY. Association of interleukin-1 polymorphisms with periodontal disease. *J Periodontol*. Feb 2005;76(2):234-43. doi:10.1902/jop.2005.76.2.234

14. Lopez NJ, Valenzuela CY, Jara L. Interleukin-1 gene cluster polymorphisms associated with periodontal disease in type 2 diabetes. *J Periodontol*. Oct 2009;80(10):1590-8. doi:10.1902/jop.2009.090134

15. Puri K, Chhokra M, Dodwad V, Puri N. Association of interleukin-1 alpha (-889) gene polymorphism in patients with generalized aggressive and chronic periodontitis. *Dent Res J (Isfahan)*. Jan-Feb 2015;12(1):76-82. doi:10.4103/1735-3327.150338

16. Rogers MA, Figliomeni L, Baluchova K, et al. Do interleukin-1 polymorphisms predict the development of periodontitis or the success of dental implants? *J Periodontal Res*. Feb 2002;37(1):37-41. doi:10.1034/j.1600-0765.2002.00651.x

17. Schulz S, Stein JM, Altermann W, et al. Single nucleotide polymorphisms in interleukin-1gene cluster and subgingival colonization with Aggregatibacter actinomycetemcomitans in patients with aggressive periodontitis. *Human Immunology*. 2011;72(10):940-946.

18. Shirodaria S, Smith J, McKay IJ, Kennett CN, Hughes FJ. Polymorphisms in the IL-1A gene are correlated with levels of interleukin-1alpha protein in gingival crevicular fluid of teeth with severe periodontal disease. *J Dent Res*. Nov 2000;79(11):1864-9. doi:10.1177/00220345000790110801

19. Trevilatto PC, de Souza Pardo AP, Scarel-Caminaga RM, et al. Association of IL1 gene polymorphisms with chronic periodontitis in Brazilians. *Arch Oral Biol*. Jan 2011;56(1):54-62. doi:10.1016/j.archoralbio.2010.09.004

20. Lavu V, Venkatesan V, Venkata Kameswara Subrahmanya Lakkakula B, Venugopal P, Paul SF, Rao SR. Polymorphic regions in the interleukin-1 gene and susceptibility to chronic periodontitis: a genetic association study. *Genet Test Mol Biomarkers*. Apr 2015;19(4):175-81. doi:10.1089/gtmb.2014.0275

21. Zuccarello D, Bazzato MF, Ferlin A, et al. Role of familiarity versus interleukin-1 genes cluster polymorphisms in chronic periodontitis. *Gene*. Feb 10 2014;535(2):286-9. doi:10.1016/j.gene.2013.11.016

22. Borilova Linhartova P, Poskerova H, Tomandlova M, et al. Interleukin-1 gene variability and plasma levels in Czech patients with chronic periodontitis and diabetes mellitus. *International journal of dentistry*. 2019;2019

23. Dominguez-Perez RA, Loyola-Rodriguez JP, Abud-Mendoza C, Alpuche-Solis AG, Ayala-Herrera JL, Martinez-Martinez RE. Association of cytokines polymorphisms with chronic peridontitis and rheumatoid arthritis in a Mexican population. *Acta Odontol Scand*. May 2017;75(4):243-248. doi:10.1080/00016357.2017.1280846

24. Majumder P, Panda SK, Ghosh S, Dey SK. Interleukin gene polymorphisms in chronic periodontitis: A case-control study in the Indian population. *Arch Oral Biol*. May 2019;101:156-164. doi:10.1016/j.archoralbio.2019.03.015

25. Mesa F, Lanza E, Garcia L, Marfil-Alvarez R, Magan-Fernandez A. Polymorphism IL-1RN rs419598 reduces the susceptibility to generalized periodontitis in a population of European descent. *PLoS One*. 2017;12(10):e0186366. doi:10.1371/journal.pone.0186366

26. Wagner J, Kaminski WE, Aslanidis C, et al. Prevalence of OPG and IL-1 gene polymorphisms in chronic periodontitis. *J Clin Periodontol*. Oct 2007;34(10):823-7. doi:10.1111/j.1600-051X.2007.01132.x
