# Supplementary material for: A Systematic Review and Meta-Analysis on Multiple Cytokine Gene Polymorphisms in the Pathogenesis of Periodontitis
Source: Front Immunol. 2022 Jan 3;12:713198. doi: 10.3389/fimmu.2021.713198 (PMC8761621; doi:10.3389/fimmu.2021.713198)
Supplement: Supplementary file 7 [file Table_7.docx]

Table S7. The association of IL-1beta polymorphism with chronic periodontitis.

**3954 C>T**

| Authors (years) | Country |  | Cases |  |  | Controls |  |  | |
| --- | --- | --- | --- | --- | --- | --- | --- | --- | --- |
| method | Value |  |  |  |  |  |  |  | |
| of p |  |  |  |  |  |  |  |  | |
|  |  | CC | CT | TT | CC | CT | TT |  | |
| Agrawal et al. 2006 | India | 16 | 49 | 25 | 12 | 15 | 3 | ^12^ | |
| AI-Hebshi et al. 2012 | Yemen | 8 | 20 | 12 | 13 | 23 | 4 | ^13^ | |
| Anusaksathien et al. 2003 | Asian | 24 | 2 | 0 | 42 | 1 | 0 | ^14^ | |
| Ayazi et al. 2013 | Caucasian | 10 | 0 | 16 | 14 | 7 | 4 | ^15^ | |
| Archana et al. 2012 | India | 16 | 29 | 0 | 14 | 1 | 0 | ^16^ | |
| Brett et al. 2005 | Caucasians | 28 | 9 | 12 | 58 | 32 | 8 | ^17^ |  |
| Droidzik et al. 2006 | Caucasians | 15 | 5 | 0 | 31 | 19 | 2 | ^18^ |  |
| Duan et al. 2002 | China | 23 | 7 | 0 | 90 | 4 | 0 | ^19^ | |
| Ebadian et al. 2013 | Caucasian | 53 | 48 | 32 | 21 | 0 | 24 | ^20^ | |
| Gayathri et al. 2011 | India | 34 | 16 | 1 | 28 | 24 | 0 | ^21^ | |
| Gonzales et al. 2003 | Caucasians | 10 | 4 | 2 | 9 | 3 | 2 | ^22^ | |
| Gonzales et al. 2003 | Caucasians | 15 | 10 | 3 | 17 | 12 | 4 |  | |
| Guzeldemir et al. 2008 | Caucasians | 19 | 11 | 1 | 0 | 7 | 24 | ^23^ | |
| Havemose et al. 2007 | Caucasians | 9 | 7 | 2 | 16 | 9 | 0 | ^24^ | |
| Havemose et al. 2007 | Caucasians | 19 | 7 | 1 | 16 | 9 | 0 |  | |
| Huang et al. 2004 | China | 151 | 31 | 0 | 85 | 4 | 0 | ^25^ | |
| Kaarthikeyan et al. 2009 | India | 21 | 9 | 0 | 24 | 6 | 1 | ^26^ | |
| Karasneh et al. 2011 | Jordan | 47 | 44 | 9 | 41 | 33 | 6 | ^27^ | |
| Kobayashi et al. 2009 | Japan | 111 | 6 | 0 | 102 | 6 | 0 | ^28^ | |
| Lin et al. 2003 | China | 89 | 35 | 0 | 148 | 24 | 0 | ^29^ | |
| Ma et al. 2011 | China | 55 | 21 | 20 | 77 | 17 | 10 | ^30^ | |
| Masamatti et al. 2012 | India | 14 | 8 | 7 | 22 | 5 | 3 | ^31^ | |
| Moreira et al. 2005 | Mixed | 46 | 31 | 34 | 12 | 0 | 24 | ^32^ | |
| Prakash et al. 2010 | Jordan | 47 | 44 | 9 | 41 | 33 | 6 | ^31^ | |
| Poulsen et al. 2007 | Caucasians | 19 | 7 | 1 | 16 | 9 | 0 | ^24^ | |
| Poulsen et al. 2007 | Caucasians | 9 | 7 | 2 | 16 | 9 | 0 |  | |
| Quappe et al. 2004 | Mixed | 25 | 11 | 0 | 63 | 10 | 2 | ^33^ | |
| Sakellari et al. 2006 | Caucasians | 26 | 16 | 4 | 50 | 35 | 5 | ^34^ | |
| Scapoli et al. 2005 | Caucasians | 24 | 15 | 1 | 42 | 43 | 11 | ^35^ | |
| Schulz et al. 2011 | Caucasian | 51 | 25 | 6 | 50 | 33 | 5 | ^36^ | |
| Shete et al. 2010 | India | 40 | 3 | 0 | 75 | 25 | 1 | ^37^ | |
| Shibani et al. 2011 | Caucasian | 15 | 14 | 3 | 22 | 8 | 5 | ^38^ | |
| Tai et al. 2002 | Asian | 45 | 2 | 0 | 88 | 13 | 0 | ^39^ | |
| Tian et al. 2006 | China | 32 | 4 | 0 | 36 | 0 | 0 | ^40^ | |
| Viicel et al. 2013 | Caucasian | 29 | 19 | 8 | 32 | 9 | 6 |  | |
| Yang et al. 2011 | China | 162 | 34 | 19 | 125 | 62 | 32 | ^41^ | |
| Zhong et al. 2002 | China | 90 | 38 | 5 | 70 | 17 | 5 | ^42^ | |

**-511C>T**

|  | CASE |  |  | CONTROL |  |  |  |  |
| --- | --- | --- | --- | --- | --- | --- | --- | --- |
|  | CC | Ct | Tt | CC | CT | TT |  |  |
| Fan et al. 2009 | 41 | 78 | 58 | 36 | 67 | 36 | ^43^ |  |
| Huang et al. 2004 | 25 | 58 | 99 | 21 | 49 | 19 | ^25^ |  |
| Li et al. 2004 | 33 | 55 | 34 | 28 | 44 | 23 | ^44^ |  |
| Li et al. 2010 | 16 | 36 | 28 | 34 | 26 | 20 | ^45^ |  |
| Loo et al. 2012 | 61 | 251 | 128 | 179 | 357 | 314 | ^46^ |  |
| Yang et al. 2008 | 11 | 20 | 13 | 18 | 21 | 10 | ^47^ |  |
| Zhang et al. 2009 | 24 | 54 | 25 | 38 | 61 | 27 | ^48^ |  |
| Zhang et al. 2004 | 36 | 52 | 40 | 37 | 53 | 40 | ^49^ |  |
| Gore et al. 1998 | 13 | 15 | 4 | 13 | 16 | 3 | ^50^ |  |
| Amirisetty et al. 2014 | 9 | 6 | 8 | 6 | 17 | 14 | ^51^ |  |
| Atanasovska et al. 2013 | 42 | 60 | 12 | 143 | 118 | 40 | ^52^ |  |
| Hao et al. 2013 | 45 | 54 | 32 | 284 | 518 | 217 | ^54^ |  |
| Tanaka et al. 2014 | 45 | 54 | 32 | 284 | 518 | 217 | ^55^ |  |
| References |  |  |  |  |  |  |  |  |

1. da Silva FRP, Vasconcelos A, de Carvalho Franca LF, Di Lenardo D, Nascimento HMS, Vasconcelos DFP. Association between the rs1143634 polymorphism in interleukin-1B and chronic periodontitis: Results from a meta-analysis composed by 54 case/control studies. *Gene*. Aug 20 2018;668:97-106. doi:10.1016/j.gene.2018.05.067

2. Chen YJ, Han Y, Mao M, Tan YQ, Leng WD, Zeng XT. Interleukin-1beta rs1143634 polymorphism and aggressive periodontitis susceptibility: a meta-analysis. *Int J Clin Exp Med*. 2015;8(2):2308-16.

3. Deng JS, Qin P, Li XX, Du YH. Association between interleukin-1beta C (3953/4)T polymorphism and chronic periodontitis: evidence from a meta-analysis. *Hum Immunol*. Mar 2013;74(3):371-8. doi:10.1016/j.humimm.2012.11.018

4. Hong SJ, Kang SW, Kim SK, Kim YS, Ban JY. Lack of Association between Interleukin-1beta Gene Polymorphism (rs16944) and Chronic Periodontitis: From a Case-Control Studies to an Updated Meta-Analysis. *Dis Markers*. 2018;2018:8287026. doi:10.1155/2018/8287026

5. Hu YY, Liu JH, Jiang GB, Yuan RX, Niu YM, Shen M. Association between Interleukin-1beta Gene -511C>T/+3954C>T Polymorphisms and Aggressive Periodontitis Susceptibility: Evidence from a Meta-Analysis. *Med Sci Monit*. Jun 3 2015;21:1617-24. doi:10.12659/MSM.894402

6. Karimbux NY, Saraiya VM, Elangovan S, et al. Interleukin-1 gene polymorphisms and chronic periodontitis in adult whites: a systematic review and meta-analysis. *J Periodontol*. Nov 2012;83(11):1407-19. doi:10.1902/jop.2012.110655

7. Ma L, Chu WM, Zhu J, Wu YN, Wang ZL. Interleukin-1beta (3953/4) C-->T polymorphism increases the risk of chronic periodontitis in Asians: evidence from a meta-analysis of 20 case-control studies. *Arch Med Sci*. Apr 25 2015;11(2):267-73. doi:10.5114/aoms.2015.50961

8. Salles AG, Antunes LAA, Kuchler EC, Antunes LS. Association between Apical Periodontitis and Interleukin Gene Polymorphisms: A Systematic Review and Meta-analysis. *J Endod*. Mar 2018;44(3):355-362. doi:10.1016/j.joen.2017.11.001

9. Wang HF, He FQ, Xu CJ, et al. Association between the interleukin-1beta C-511T polymorphism and periodontitis: a meta-analysis in the Chinese population. *Genet Mol Res*. Feb 23 2017;16(1)doi:10.4238/gmr16019315

10. Zeng XT, Liu DY, Kwong JS, Leng WD, Xia LY, Mao M. Meta-Analysis of Association Between Interleukin-1beta C-511T Polymorphism and Chronic Periodontitis Susceptibility. *J Periodontol*. Jun 2015;86(6):812-9. doi:10.1902/jop.2015.140698

11. Stadler AF, Angst PD, Arce RM, Gomes SC, Oppermann RV, Susin C. Gingival crevicular fluid levels of cytokines/chemokines in chronic periodontitis: a meta-analysis. *J Clin Periodontol*. Sep 2016;43(9):727-45. doi:10.1111/jcpe.12557

12. Agrawal AA, Kapley A, Yeltiwar RK, Purohit HJ. Assessment of single nucleotide polymorphism at IL‐1A+ 4845 and IL‐1B+ 3954 as genetic susceptibility test for chronic periodontitis in Maharashtrian ethnicity. *Journal of periodontology*. 2006;77(9):1515-1521.

13. Al-Hebshi NN, Shamsan A-aA, Al-Ak'hali MS. Interleukin-1 two-locus haplotype is strongly associated with severe chronic periodontitis among Yemenis. *Molecular biology international*. 2012;2012

14. Anusaksathien O, Sukboon A, Sitthiphong P, Teanpaisan R. Distribution of interleukin-1beta(+3954) and IL-1alpha(-889) genetic variations in a Thai population group. *J Periodontol*. Dec 2003;74(12):1796-802. doi:10.1902/jop.2003.74.12.1796

15. Ayazi G, Pirayesh M, Yari K. Analysis of interleukin-1beta gene polymorphism and its association with generalized aggressive periodontitis disease. *DNA Cell Biol*. Jul 2013;32(7):409-13. doi:10.1089/dna.2012.1905

16. Archana P, Salman AA, Kumar TS, Saraswathi P, Panishankar K, Kumarasamy P. Association between interleukin-1 gene polymorphism and severity of chronic periodontitis in a south Indian population group. *Journal of Indian Society of Periodontology*. 2012;16(2):174.

17. Brett PM, Zygogianni P, Griffiths GS, et al. Functional gene polymorphisms in aggressive and chronic periodontitis. *J Dent Res*. Dec 2005;84(12):1149-53. doi:10.1177/154405910508401211

18. Droździk A, Kurzawski M, Safronow K, Banach J. Polymorphism in interleukin-1beta gene and the risk of periodontitis in a Polish population. *Advances in medical sciences*. 2006;51:13-17.

19. Duan H, Zhang J, Zhang Y. The association between IL-1 gene polymorphisms and susceptibility to severe periodontitis. *Hua xi kou qiang yi xue za zhi= Huaxi kouqiang yixue zazhi= West China journal of stomatology*. 2002;20(1):48-51.

20. Ebadian AR, Radvar M, Afshari JT, et al. Gene polymorphisms of TNF-α and IL-1β are not associated with generalized aggressive periodontitis in an Iranian subpopulation. *Iranian Journal of Allergy, Asthma and Immunology*. 2013:345-351.

21. Gayathri R, Saadi AV, Bhat KM, Bhat SG, Satyamoorthy K. Allele, genotype, and composite genotype effects of IL-1A +4845 and IL-1B +3954 polymorphisms for chronic periodontitis in an Indian population. *Indian J Dent Res*. Jul-Aug 2011;22(4):612. doi:10.4103/0970-9290.90323

22. Gonzales JR, Michel J, Rodriguez EL, Herrmann JM, Bodeker RH, Meyle J. Comparison of interleukin-1 genotypes in two populations with aggressive periodontitis. *Eur J Oral Sci*. Oct 2003;111(5):395-9. doi:10.1034/j.1600-0722.2003.00071.x

23. Guzeldemir E, Gunhan M, Ozcelik O, Tastan H. Interleukin-1 and tumor necrosis factor-alpha gene polymorphisms in Turkish patients with localized aggressive periodontitis. *J Oral Sci*. Jun 2008;50(2):151-9. doi:10.2334/josnusd.50.151

24. Havemose‐Poulsen A, Sørensen LK, Bendtzen K, Holmstrup P. Polymorphisms within the IL‐1 gene cluster: Effects on cytokine profiles in peripheral blood and whole blood cell cultures of patients with aggressive periodontitis, juvenile idiopathic arthritis, and rheumatoid arthritis. *Journal of periodontology*. 2007;78(3):475-492.

25. Huang H, Zhang J. Investigation on the association of interleukin-1 genotype polymorphism with chronic periodontitis. *Hua xi kou qiang yi xue za zhi= Huaxi kouqiang yixue zazhi= West China journal of stomatology*. 2004;22(5):415-419.

26. Kaarthikeyan G, Jayakumar N, Padmalatha O, Sheeja V. Sankari; and M, Anandan B.(2009). Analysis of the association between interleukin-1beta (+ 3954) gene polymorphism and chronic periodontitis in a sample of the south Indian population. *Indian J Dent Res*. 20:37-40.

27. Karasneh JA, Ababneh KT, Taha AH, Al-Abbadi MS, Ollier WE. Investigation of the interleukin-1 gene cluster polymorphisms in Jordanian patients with chronic and aggressive periodontitis. *Arch Oral Biol*. Mar 2011;56(3):269-76. doi:10.1016/j.archoralbio.2010.10.001

28. Kobayashi T, Murasawa A, Ito S, et al. Cytokine gene polymorphisms associated with rheumatoid arthritis and periodontitis in Japanese adults. *J Periodontol*. May 2009;80(5):792-9. doi:10.1902/jop.2009.080573

29. Lin L, Pan Y, Yin L-Y. Study on the correlation of cytokine gene polymorphism with chronic periodontitis. *Shanghai kou qiang yi xue= Shanghai journal of stomatology*. 2003;12(6):456-459.

30. Ma M, Li G, Han C, Huang Y. Correlation study on polymorphisms of the Interleukin-1 and tumor necrosis factoralpha gene in Hui patients with chronic periodontitis in Ningxia. *Journal of Modern Stomatology*. 2011;25:94-97.

31. Masamatti SS, Kumar A, Baron TK, Mehta DS, Bhat K. Evaluation of interleukin -1B (+3954) gene polymorphism in patients with chronic and aggressive periodontitis: A genetic association study. *Contemp Clin Dent*. Apr 2012;3(2):144-9. doi:10.4103/0976-237X.96815

32. Moreira PR, De Sá AR, Xavier GM, et al. A functional interleukin‐1β gene polymorphism is associated with chronic periodontitis in a sample of Brazilian individuals. *Journal of periodontal research*. 2005;40(4):306-311.

33. Quappe L, Jara L, Lopez NJ. Association of interleukin-1 polymorphisms with aggressive periodontitis. *J Periodontol*. Nov 2004;75(11):1509-15. doi:10.1902/jop.2004.75.11.1509

34. Sakellari D, Katsares V, Georgiadou M, Kouvatsi A, Arsenakis M, Konstantinidis A. No correlation of five gene polymorphisms with periodontal conditions in a Greek population. *Journal of clinical periodontology*. 2006;33(11):765-770.

35. Scapoli C, Trombelli L, Mamolini E, Collins A. Linkage disequilibrium analysis of case-control data: an application to generalized aggressive periodontitis. *Genes Immun*. Feb 2005;6(1):44-52. doi:10.1038/sj.gene.6364152

36. Schulz S, Stein JM, Altermann W, et al. Single nucleotide polymorphisms in interleukin-1gene cluster and subgingival colonization with Aggregatibacter actinomycetemcomitans in patients with aggressive periodontitis. *Hum Immunol*. Oct 2011;72(10):940-6. doi:10.1016/j.humimm.2011.05.009

37. Shete AR, Joseph R, Vijayan NN, Srinivas L, Banerjee M. Association of Single Nucleotide Gene Polymorphism at Interleukin‐1β+ 3954,− 511, and− 31 in Chronic Periodontitis and Aggressive Periodontitis in Dravidian Ethnicity. *Journal of periodontology*. 2010;81(1):62-69.

38. Shibani K, Shhab R, Khattab R. Analysis of IL-1alpha(-889) and IL-1B(+3953) Gene Polymorphism in Syrian Patients with Aggressive Periodontitis: A Pilot Study. *ISRN Dent*. 2011;2011:682564. doi:10.5402/2011/682564

39. Tai H, Endo M, Shimada Y, et al. Association of interleukin-1 receptor antagonist gene polymorphisms with early onset periodontitis in Japanese. *J Clin Periodontol*. Oct 2002;29(10):882-8. doi:10.1034/j.1600-051x.2002.291002.x

40. Tian Y-G, Pan Y-P, Lin L, Zhang D-M, Zhao J. The relationship of IL-1beta expressed in buccal cells and the polymorphisms of IL-1beta (+ 3953) with chronic periodontitis. *Shanghai kou qiang yi xue= Shanghai journal of stomatology*. 2006;15(5):456-460.

41. Yang L, Xie X, Ma L, Liu Z, Pan Y, Liu Y. A study of association between the interleukin-1 single nucleotide polymorphism and risk of chronic periodontitis among the Hui and Dongxiang minorities in Gansu province. *Hua xi kou qiang yi xue za zhi= Huaxi kouqiang yixue zazhi= West China journal of stomatology*. 2011;29(4):365-368.

42. Zhong L, Zhang Y, Zhang J, Yang A, Huang H. [The association of interleukin-1 gene polymorphisms with the susceptibility to chronic periodontitis in Uighur]. *Zhonghua Yi Xue Yi Chuan Xue Za Zhi*. Oct 2002;19(5):405-8.

43. Fan W. Investigation on polymorphisms of interleukin-6 and interleukin-1 genes as the risk factors for coronary heart disease and chronic periodontitis. *Southern Medical University*. 2009;

44. Li QY, Zhao HS, Meng HX, et al. Association analysis between interleukin‐1 family polymorphisms and generalized aggressive periodontitis in a Chinese population. *Journal of periodontology*. 2004;75(12):1627-1635.

45. Li N, Song J, Su J, Li K, Shi T, Gao X. Association of interleukin-1 beta 511C/T gene polymorphism with dibetes and chronic periodontitis. *Shangdong Yi Yao*. 2010;50:71-72.

46. Loo WT, Fan C-b, Bai L-j, et al. Gene polymorphism and protein of human pro-and anti-inflammatory cytokines in Chinese healthy subjects and chronic periodontitis patients. BioMed Central; 2012:1-10.

47. Yang K. Interleukin-1 genotype and the association between chronic periodontitis and coronary heart disease. *Urumqi Xinjiang Medical University*. 2008;

48. Zhang L. The association of interleukin-1 gene polymorphisms with the susceptibility to chronic periodontitis in Dongxiang populations from Northwestern China. *Lanzhou University*. 2009;

49. Zhang Y, Zhong L, Gulibositan D, Nie J. Interleukin-1 B-511 genotype and the association between chronic periodontitis and coronary heart disease. *Xinjiang Yi Ke Da Xue Xue Bao*. 2004;27:202-204.

50. Gore EA, Sanders JJ, Pandey JP, Palesch Y, Galbraith GM. Interleukin-1beta+3953 allele 2: association with disease status in adult periodontitis. *J Clin Periodontol*. Oct 1998;25(10):781-5. doi:10.1111/j.1600-051x.1998.tb02370.x

51. Amirisetty R, Patel RP, Das S, Saraf J, Jyothy A, Munshi A. Interleukin 1beta (+3954, -511 and -31) polymorphism in chronic periodontitis patients from North India. *Acta Odontol Scand*. Jul 2015;73(5):343-7. doi:10.3109/00016357.2014.961958

52. Atanasovska-Stojanovska A, Popovska M, Trajkov D, Spiroski M. IL1 cluster gene polymorphisms in Macedonian patients with chronic periodontitis. *Bratisl Lek Listy*. 2013;114(7):380-5. doi:10.4149/bll_2013_080

53. Braosi AP, de Souza CM, Luczyszyn SM, et al. Analysis of IL1 gene polymorphisms and transcript levels in periodontal and chronic kidney disease. *Cytokine*. Oct 2012;60(1):76-82. doi:10.1016/j.cyto.2012.06.006

54. Hao L, Li JL, Yue Y, et al. Application of interleukin-1 genes and proteins to monitor the status of chronic periodontitis. *Int J Biol Markers*. Apr 23 2013;28(1):92-9. doi:10.5301/jbm.5000013

55. Tanaka K, Miyake Y, Hanioka T, Arakawa M. Relationship between IL1 gene polymorphisms and periodontal disease in Japanese women. *DNA and cell biology*. 2014;33(4):227-233.
