# Supplementary material for: A Systematic Review and Meta-Analysis on Multiple Cytokine Gene Polymorphisms in the Pathogenesis of Periodontitis
Source: Front Immunol. 2022 Jan 3;12:713198. doi: 10.3389/fimmu.2021.713198 (PMC8761621; doi:10.3389/fimmu.2021.713198)
Supplement: Supplementary file 8 [file Table_8.docx]

Table S8. The association of IL-6 -174G/C polymorphism with chronic periodontitis.

| Authors and years | Ethnicity | Cases /controls | Case *GG* | *GC* | *CC* | Control *GG* | *GC* | *CC* | HWE  P value |  |
| --- | --- | --- | --- | --- | --- | --- | --- | --- | --- | --- |
| Gabriela Teixeira F et al.2014 | Brazilian | 134/196 | 102 | 23 | 9 | 136 | 54 | 6 | 0.82 | ^11^ |
| Stefani FA et al.2013 | Brazilian | 21/21 | 12 | 8 | 1 | 11 | 8 | 2 | 0.76 | ^12^ |
| Casado PL et al.2013 | Brazilian | 43/103 | 24 | 16 | 3 | 53 | 42 | 8 | 0.94 | ^13^ |
| Moreira PR et al.2007 | Brazilian | 261/106 | 94 | 42 | 11 | 60 | 35 | 11 | 0.10 | ^9^ |
| Costa AM et al.2010 | Brazilian | 38/27 | 15 | 2 | 0 | 12 | 12 | 3 | 1.00 | ^14^ |
| Erciyas K et al. 2009 | Turkish | 35/85 | 22 | 12 | 1 | 49 | 31 | 5 | 0.97 | ^15^ |
| Nibali Let al. 2009 | Black | 90/45 | 81 | 9 | 0 | 38 | 7 | 0 | 0.57 | ^10^ |
| Nibali Let al. 2009 | Asian | 85/29 | 68 | 15 | 2 | 22 | 6 | 1 | 0.48 | ^10^ |
| Kalburgi NB et al.2010 | Indian | 15/15 | 10 | 2 | 3 | 2 | 4 | 9 | 0.22 | ^16^ |
| Kavitha Let al.2017 | Indian | 60/30 | 47 | 11 | 2 | 29 | 1 | 0 | 0.93 | ^17^ |
| Fan WH et al.2011 | Chinese Han | 178/130 | 184 | 95 | 12 | 95 | 32 | 3 | 0.88 | ^18^ |
| Holla LI et al.2004 | Caucasian | 148/107 | 43 | 71 | 34 | 37 | 53 | 17 | 0.78 | ^19^ |
| Toker H et al.2017 | Caucasian | 45/38 | 25 | 12 | 8 | 12 | 13 | 13 | 0.05 | ^20^ |
| Pirim Gorgun E et al.2017 | Caucasian | 53/50 | 31 | 18 | 4 | 20 | 14 | 16 | 0.18 | ^21^ |
| Scapoli Let al.2015 | Caucasian | 285/215 | 131 | 127 | 26 | 77 | 92 | 42 | 0.13 | ^22^ |
| Ianni Met al.2013 | Caucasian | 77/278 | 40 | 25 | 12 | 119 | 125 | 34 | 0.89 | ^23^ |
| Trevilatto PC et al.2007 | Caucasian | 48/36 | 12 | 12 | 0 | 12 | 21 | 3 | 0.14 | ^7^ |
|  |  |  | 17 | 3 | 4 |  |  |  |  |  |

References

1. Ioannidou E, Kao D, Chang N, Burleson J, Dongari‐Bagtzoglou A. Elevated serum interleukin‐6 (IL‐6) in solid‐organ transplant recipients is positively associated with tissue destruction and IL‐6 gene expression in the periodontium. *Journal of periodontology*. 2006;77(11):1871-1878.

2. Blach A, Franek E, Witula A, et al. The influence of chronic periodontitis on serum TNF-alpha, IL-6 and hs-CRP concentrations, and function of graft and survival of kidney transplant recipients. *Clin Transplant*. Mar-Apr 2009;23(2):213-9. doi:10.1111/j.1399-0012.2008.00931.x

3. Shaqman M, Ioannidou E, Burleson J, Hull D, Dongari-Bagtzoglou A. Periodontitis and inflammatory markers in transplant recipients. *J Periodontol*. May 2010;81(5):666-72. doi:10.1902/jop.2010.090570

4. Gürkan A, Becerik S, Öztürk VÖ, Atmaca H, Atilla G, Emingil G. Interleukin‐6 Family of Cytokines in Crevicular Fluid of Renal Transplant Recipients With and Without Cyclosporine A–Induced Gingival Overgrowth. *Journal of periodontology*. 2015;86(9):1069-1077.

5. Abdolsamadi HR, Vahedi M, Esmaeili F, Nazari S, Abdollahzadeh S. Serum interleukin-6 as a serologic marker of chronic periapical lesions: a case-control study. *Journal of dental research, dental clinics, dental prospects*. 2008;2(2):43.

6. Garrido M, Cárdenas AM, Astorga J, et al. Elevated systemic inflammatory burden and cardiovascular risk in young adults with endodontic apical lesions. *Journal of endodontics*. 2019;45(2):111-115.

7. Trevilatto P, Scarel‐Caminaga R, de Brito Jr R, De Souza A, Line S. Polymorphism at position− 174 of IL‐6 gene is associated with susceptibility to chronic periodontitis in a Caucasian Brazilian population. *Journal of clinical periodontology*. 2003;30(5):438-442.

8. Wohlfahrt JC, Wu T, Hodges JS, Hinrichs JE, Michalowicz BS. No association between selected candidate gene polymorphisms and severe chronic periodontitis. *Journal of periodontology*. 2006;77(3):426-436.

9. Moreira P, Lima P, Sathler K, et al. Interleukin‐6 expression and gene polymorphism are associated with severity of periodontal disease in a sample of Brazilian individuals. *Clinical & Experimental Immunology*. 2007;148(1):119-126.

10. Nibali L, D’aiuto F, Donos N, et al. Association between periodontitis and common variants in the promoter of the interleukin-6 gene. *Cytokine*. 2009;45(1):50-54.

11. Gabriela Teixeira F, Mendonça SA, Menezes Oliveira K, et al. Interleukin-6 c.-174G> C polymorphism and periodontitis in a Brazilian population. *Molecular biology international*. 2014;2014

12. Stefani FA, Viana MB, Dupim AC, et al. Expression, polymorphism and methylation pattern of interleukin-6 in periodontal tissues. *Immunobiology*. Jul 2013;218(7):1012-7. doi:10.1016/j.imbio.2012.12.001

13. Ladeira Casado P, Villas-Boas R, de Mello W, Leite Duarte ME, Mauro Granjeiro J. Peri-implant disease and chronic periodontitis: is interleukin-6 gene promoter polymorphism the common risk factor in a Brazilian population? *International Journal of Oral & Maxillofacial Implants*. 2013;28(1)

14. Costa A, Guimarães M, De Souza ER, Nóbrega O, Bezerra A. Interleukin‐6 (G‐174C) and tumour necrosis factor‐alpha (G‐308A) gene polymorphisms in geriatric patients with chronic periodontitis. *Gerodontology*. 2010;27(1):70-75.

15. Karaoglan I, Pehlivan S, Namiduru M, et al. TNF-alpha, TGF-beta, IL-10, IL-6 and IFN-gamma gene polymorphisms as risk factors for brucellosis. *New Microbiol*. 2009;32(2):173-8.

16. Kalburgi NB, Bhatia A, Bilichodmath S, Patil SR, Mangalekar SB, Bhat K. Interleukin-6 promoter polymorphism (-174 G/C) in Indian patients with chronic periodontitis. *J Oral Sci*. Sep 2010;52(3):431-7. doi:10.2334/josnusd.52.431

17. Kavitha L, Vijayshree Priyadharshini J, Sivapathasundharam B. Association among interleukin‐6 gene polymorphisms, type 2 diabetes mellitus, and chronic periodontitis: a pilot study. *Journal of investigative and clinical dentistry*. 2017;8(3):e12230.

18. Fan W, Liu D, Xiao L, Xie C, Sun S, Zhang J. Coronary heart disease and chronic periodontitis: is polymorphism of interleukin‐6 gene the common risk factor in a Chinese population? *Oral Diseases*. 2011;17(3):270-276.

19. Holla LI, Fassmann A, Stejskalová A, Znojil V, Vaněk J, Vacha J. Analysis of the interleukin‐6 gene promoter polymorphisms in Czech patients with chronic periodontitis. *Journal of periodontology*. 2004;75(1):30-36.

20. Toker H, Görgün EP, Korkmaz EM. Analysis of IL-6, IL-10 and NF-ΚB gene polymorphisms in aggressive and chronic periodontitis. *Central European journal of public health*. 2017;25(2):157-162.

21. PIRIM GORGUN E, Toker H, Korkmaz EM, Poyraz O. IL-6 and IL-10 gene polymorphisms in patients with aggressive periodontitis: effects on GCF, serum and clinic parameters. *Brazilian oral research*. 2017;31

22. Scapoli L, Girardi A, Palmieri A, et al. Interleukin-6 gene polymorphism modulates the risk of periodontal diseases. *J Biol Regul Homeost Agents*. 2015;29(3 Suppl 1):111-6.

23. Ianni M, Bruzzesi G, Pugliese D, et al. Variations in inflammatory genes are associated with periodontitis. *Immunity & Ageing*. 2013;10(1):1-8.

24. Xiao L, Yan Y, Xie C, et al. Association among interleukin‐6 gene polymorphism, diabetes and periodontitis in a Chinese population. *Oral diseases*. 2009;15(8):547-553.
