# Supplementary material for: A Systematic Review and Meta-Analysis on Multiple Cytokine Gene Polymorphisms in the Pathogenesis of Periodontitis
Source: Front Immunol. 2022 Jan 3;12:713198. doi: 10.3389/fimmu.2021.713198 (PMC8761621; doi:10.3389/fimmu.2021.713198)
Supplement: Supplementary file 10 [file Table_10.docx]

Table S10. The association of IL-10 1082A>G polymorphism with chronic periodontitis

| Author and year | Ethnicity | Controls | Cases AA/AG/GG | Controls AA/AG/GG |  |
| --- | --- | --- | --- | --- | --- |
| Atanasovska‐Stojanovska et al. 2012 | Caucasion | 299 | 34/62/15 | 70/212/17 | ^8^ |
| Babel et al. 2006 | Caucasion | 114 | 36/82/ | 30/84/ | ^24^ |
| Berglundh et al. 2003 | Caucasion | 39 | 14/22/24 | 12/19/8 | ^25^ |
| Brett et al. 2005 | Caucasion | 92 | 18/24/13 | 28/47/17 | ^26^ |
| Chambrone et al. 2014 | Peruvian | 53 | 22/18/13 | 11/16/26 | ^27^ |
| Crena et al. 2015 | Deavidian | 45 | 34/11/1 | 27/10/8 | ^28^ |
| Gurol et al. 2011 | Turkish | 34 | 1/15/2 | 11/21/2 | ^29^ |
| Hannum et al. 2015 | Brazilian | 30 | 7/16/13 | 5/13/12 | ^30^ |
| Houshmand et al. 2013 | Iran | 30 | 22/23/7 | 3/7/20 | ^39^ |
| Hu et al. 2009 | Han | 126 | 132/13/0 | 115/11/0 | ^12^ |
| Ianni et al. 2013 | Caucasian | 470 | 39/29/7 | 168/209/93 | ^31^ |
| Jaradat et al. 2012 | Jordanian | 86 | 38/47/20 | 17/36/33 | ^13^ |
| Kobayashi et al. 2009 | Japanese | 108 | 109/8/0 | 102/6/0 | ^32^ |
| Li et al. 2009 | Han | 30 | 24/6/0 | 22/8/0 | ^14^ |
| Loo et al. 2012 | Han | 850 | 277/31/132 | 782/17/51 | ^33^ |
| Lopes et al. 2017 | Brazilian | 150 | 36/14/5 | 71/61/18 | ^15^ |
| Mellati et al. 2007 | Khorasanian | 61 | 16/27/9 | 23/31/7 | ^34^ |
| Moreira et al. 2009 | Mixed | 43 | 31/28/8 | 16/20/7 | ^35^ |
| Moudi et al. 2018 | Iranian | 100 | 34/98/78 | 38/41/21 | ^16^ |
| Reichert et al. 2008 | Caucasion | 34 | 5/17/5 | 10/18/6 | ^17^ |
| Scapoli et al. 2012 | Caucasian | 217 | 81/72/23 | 93/87/37 | ^18^ |
| Scapoli et al. 2015 | Caucasian | 201 | 117/120/43 | 76/87/38 | ^19^ |
| Scarel‐Caminaga et al. 2004 | Mixed | 43 | 34/25/8 | 17/21/5 | ^20^ |
| Silveira et al. 2016 | Brazilian | 41 | 27/25/8 | 23/14/4 | ^21^ |
| Tervonen et al. 2007 | Caucasian | 178 | 19/32/ | 54/124/ | ^36^ |
| Wang et al. 2009 | Han | 138 | 134/12/0 | 123/15/0 | ^37^ |
| Wang et al. 2012 | Han | 308 | 240/54/5 | 247/60/1 | ^38^ |

**References**

1. Li Y, Feng G, Deng Y, Song J. Contribution of Interleukin-10-592 (-590, -597) C>A Polymorphisms to Periodontitis Susceptibility: An Updated Meta-Analysis Based on 18 Case-Control Studies. *Dis Markers*. 2018;2018:2645963. doi:10.1155/2018/2645963

2. Li Y, Hu B, Feng G, et al. Association of interleukin-10-1082 (-1087) A > G polymorphisms and periodontitis risk: An updated meta-analysis based on 26 case-control studies. *Ann Hum Genet*. Sep 2019;83(5):299-309. doi:10.1111/ahg.12321

3. Mashhadiabbas F, Dastgheib SA, Hashemzehi A, et al. Association of IL-10 -1082A>G, -819C>T, and -592C>A polymorphisms with susceptibility to chronic and aggressive periodontitis: a systematic review and meta-analysis. *Inflamm Res*. Mar 5 2021;doi:10.1007/s00011-021-01448-z

4. Wang Z, Li Y, Zhou Y, Qiao Y. Association between the IL-10 rs1800872 polymorphisms and periodontitis susceptibility: A meta-analysis. *Medicine (Baltimore)*. Oct 2019;98(40):e17113. doi:10.1097/MD.0000000000017113

5. Wong HC, Ooi Y, Pulikkotil SJ, Naing C. The role of three interleukin 10 gene polymorphisms (- 1082 A > G, - 819 C > T, - 592 A > C) in the risk of chronic and aggressive periodontitis: a meta-analysis and trial sequential analysis. *BMC Oral Health*. Oct 22 2018;18(1):171. doi:10.1186/s12903-018-0637-9

6. Yang SL, Huang SJ. Interleukin-10 polymorphisms (rs1800871, rs1800872 and rs1800896) and periodontitis risk: A meta-analysis. *Arch Oral Biol*. Jan 2019;97:59-66. doi:10.1016/j.archoralbio.2018.10.012

7. Zhong Q, Ding C, Wang M, Sun Y, Xu Y. Interleukin-10 gene polymorphisms and chronic/aggressive periodontitis susceptibility: a meta-analysis based on 14 case-control studies. *Cytokine*. Oct 2012;60(1):47-54. doi:10.1016/j.cyto.2012.05.014

8. Atanasovska-Stojanovska A, Trajkov D, Popovska M, Spiroski M. IL10 -1082, IL10 -819 and IL10 -592 polymorphisms are associated with chronic periodontitis in a Macedonian population. *Hum Immunol*. Jul 2012;73(7):753-8. doi:10.1016/j.humimm.2012.04.009

9. Claudino M, Trombone AP, Cardoso CR, et al. The broad effects of the functional IL-10 promoter-592 polymorphism: modulation of IL-10, TIMP-3, and OPG expression and their association with periodontal disease outcome. *J Leukoc Biol*. Dec 2008;84(6):1565-73. doi:10.1189/jlb.0308184

10. Garlet GP, Trombone APF, Menezes R, et al. The use of chronic gingivitis as reference status increases the power and odds of periodontitis genetic studies–a proposal based in the exposure concept and clearer resistance and susceptibility phenotypes definition. *Journal of clinical periodontology*. 2012;39(4):323-332.

11. Pirim Gorgun E, Toker H, Korkmaz EM, Poyraz O. IL-6 and IL-10 gene polymorphisms in patients with aggressive periodontitis: effects on GCF, serum and clinic parameters. *Braz Oral Res*. Jan 16 2017;31:e12. doi:10.1590/1807-3107BOR-2017.vol31.0012

12. Hu KF, Huang KC, Ho YP, et al. Interleukin-10 (-592 C/A) and interleukin-12B (+16974 A/C) gene polymorphisms and the interleukin-10 ATA haplotype are associated with periodontitis in a Taiwanese population. *J Periodontal Res*. Jun 2009;44(3):378-85. doi:10.1111/j.1600-0765.2008.01116.x

13. Jaradat SM, Ababneh KT, Jaradat SA, et al. Association of interleukin-10 gene promoter polymorphisms with chronic and aggressive periodontitis. *Oral Dis*. Apr 2012;18(3):271-9. doi:10.1111/j.1601-0825.2011.01872.x

14. Li Y, Zhao H, Zhang J. Interleukin-10 gene promoter polymorphism in chinese patients with generalized aggressive periodontitis. *Journal of Dental Prevention and Treatment*. 2009;17(10):472-475.

15. Lopes CB, Barroso RFF, Burbano RMR, et al. Effect of ancestry on interleukin-10 haplotypes in chronic periodontitis. *Front Biosci (Elite Ed)*. Jun 1 2017;9:276-285. doi:10.2741/e802

16. Moudi B, Heidari Z, Mahmoudzadeh-Sagheb H, Moudi M. Analysis of interleukin-10 gene polymorphisms in patients with chronic periodontitis and healthy controls. *Dent Res J (Isfahan)*. Jan-Feb 2018;15(1):71-79. doi:10.4103/1735-3327.223614

17. Reichert S, Machulla H, Klapproth J, et al. The interleukin‐10 promoter haplotype ATA is a putative risk factor for aggressive periodontitis. *Journal of periodontal research*. 2008;43(1):40-47.

18. Scapoli L, Girardi A, Palmieri A, et al. IL6 and IL10 are genetic susceptibility factors of periodontal disease. *Dent Res J (Isfahan)*. Dec 2012;9(Suppl 2):S197-201. doi:10.4103/1735-3327.109754

19. Scapoli L, Girardi A, Palmieri A, et al. Interleukin-6 Gene Polymorphism Modulates the Risk of Periodontal Diseases. *J Biol Regul Homeost Agents*. Jul-Sep 2015;29(3 Suppl 1):111-6.

20. Scarel-Caminaga RM, Trevilatto PC, Souza AP, Brito RB, Camargo LE, Line SR. Interleukin 10 gene promoter polymorphisms are associated with chronic periodontitis. *J Clin Periodontol*. Jun 2004;31(6):443-8. doi:10.1111/j.1600-051X.2004.00500.x

21. Silveira VR, Pigossi SC, Scarel-Caminaga RM, Cirelli JA, Rego R, Nogueira NA. Analysis of polymorphisms in Interleukin 10, NOS2A, and ESR2 genes in chronic and aggressive periodontitis. *Braz Oral Res*. Oct 10 2016;30(1):e105. doi:10.1590/1807-3107BOR-2016.vol30.0105

22. Sumer AP, Kara N, Keles GC, Gunes S, Koprulu H, Bagci H. Association of interleukin-10 gene polymorphisms with severe generalized chronic periodontitis. *J Periodontol*. Mar 2007;78(3):493-7. doi:10.1902/jop.2007.060309

23. Yuhui Z, Ping H, Jing L, Jin Z. Correlation between interleukin-10 polymorphisms and susceptibility to chronic periodontitis among Uygur adults in the Moyu area. *Hua xi kou qiang yi xue za zhi= Huaxi kouqiang yixue zazhi= West China journal of stomatology*. 2017;35(5):514-519.

24. Babel N, Cherepnev G, Babel D, et al. Analysis of tumor necrosis factor-alpha, transforming growth factor-beta, interleukin-10, IL-6, and interferon-gamma gene polymorphisms in patients with chronic periodontitis. *J Periodontol*. Dec 2006;77(12):1978-83. doi:10.1902/jop.2006.050315

25. Berglundh T, Donati M, Hahn-Zoric M, Hanson LA, Padyukov L. Association of the -1087 IL 10 gene polymorphism with severe chronic periodontitis in Swedish Caucasians. *J Clin Periodontol*. Mar 2003;30(3):249-54. doi:10.1034/j.1600-051x.2003.10274.x

26. Brett PM, Zygogianni P, Griffiths GS, et al. Functional gene polymorphisms in aggressive and chronic periodontitis. *J Dent Res*. Dec 2005;84(12):1149-53. doi:10.1177/154405910508401211

27. Chambrone L, Ascarza A, Guerrero ME, et al. Association of -1082 interleukin-10 gene polymorphism in Peruvian adults with chronic periodontitis. *Med Oral Patol Oral Cir Bucal*. Nov 1 2014;19(6):e569-73. doi:10.4317/medoral.19823

28. Crena J, Sangeetha Subramanian DJV, Gnana PPS, Ramanathan A. Single nucleotide polymorphism at− 1087 locus of interleukin-10 gene promoter is associated with severe chronic periodontitis in nonsmoking patients. *European journal of dentistry*. 2015;9(3):387.

29. Gurol C, Kazazoglu E, Dabakoglu B, Korachi M. A comparative study of the role of cytokine polymorphisms interleukin-10 and tumor necrosis factor alpha in susceptibility to implant failure and chronic periodontitis. *Int J Oral Maxillofac Implants*. Sep-Oct 2011;26(5):955-60.

30. Hannum R, Godoy FR, Cruz ASd, et al. Lack of association between IL-10-1082G/A polymorphism and chronic periodontal disease in adults. 2015;

31. Ianni M, Bruzzesi G, Pugliese D, et al. Variations in inflammatory genes are associated with periodontitis. *Immun Ageing*. Oct 1 2013;10(1):39. doi:10.1186/1742-4933-10-39

32. Kobayashi T, Murasawa A, Ito S, et al. Cytokine gene polymorphisms associated with rheumatoid arthritis and periodontitis in Japanese adults. *J Periodontol*. May 2009;80(5):792-9. doi:10.1902/jop.2009.080573

33. Loo WT, Fan C-b, Bai L-j, et al. Gene polymorphism and protein of human pro-and anti-inflammatory cytokines in Chinese healthy subjects and chronic periodontitis patients. BioMed Central; 2012:1-10.

34. Mellati E, Arab HR, Tavakkol-Afshari J, Ebadian AR, Radvar M. Analysis of -1082 IL-10 gene polymorphism in Iranian patients with generalized aggressive periodontitis. *Med Sci Monit*. Nov 2007;13(11):CR510-514.

35. Moreira P, Costa J, Gomez R, Gollob K, Dutra W. TNFA and IL10 gene polymorphisms are not associated with periodontitis in Brazilians. *The Open Dentistry Journal*. 2009;3:184.

36. Tervonen T, Raunio T, Knuuttila M, Karttunen R. Polymorphisms in the CD14 and IL-6 genes associated with periodontal disease. *J Clin Periodontol*. May 2007;34(5):377-83. doi:10.1111/j.1600-051X.2007.01067.x

37. Wang C, Zhang J, Zhao H, Fan W, Xiao L, Xie B. Correlation of interleukin‐10‐1082 G/A single nucleotide polymorphism to the risk of severe chronic periodontitis in Chinese: A case‐control study. *Journal of Dental Prevention and Treatment*. 2009;17:354-356.

38. Wang J, Yuan W, Fu F, Yang J, Tang F. A case‐control study on Interleukin‐10 genetic polymorphisms and susceptibility to chronic periodontitis. *International Journal of Epidemiology of Infectious Diseases*. 2012;39:306-309.

39. Heidari Z, Hooshmand B, Hajilooi M, Kadkhodazadeh M. Study of IL-10, IL-18 polymorphisms in the sulfur agent patients with and without periodontitis. *Journal of Advanced Periodontology & Implant Dentistry*. 2018;5(1):23-28.
