# Supplementary material for: A Systematic Review and Meta-Analysis on Multiple Cytokine Gene Polymorphisms in the Pathogenesis of Periodontitis
Source: Front Immunol. 2022 Jan 3;12:713198. doi: 10.3389/fimmu.2021.713198 (PMC8761621; doi:10.3389/fimmu.2021.713198)
Supplement: Supplementary file 11 [file Table_11.docx]

Table S11. The association of IL-17 rs2275913 polymorphism with chronic periodontitis

| Study or Subgroup | Experimental Events Total | | Control  Events Total Weight | | | Odds Ratio  M-H, Random, 95% CI | |  | | | |
| --- | --- | --- | --- | --- | --- | --- | --- | --- | --- | --- | --- |
|  | Events | Total |  |  |  |  | | References | |  |  |
| Borilova et al. 2016 | 161 | 488 | 107 | 308 | 0.92 [0.68, 1.25] | | ^3^ | |  |  |  |
| Chaudhari et al. 2015 | 48 | 70 | 21 | 70 | 5.09 [2.48, 10.44] | | ^4^ | |  |  |  |
| Correa et al. 2012 | 39 | 60 | 20 | 60 | 3.71 [1.75, 7.90] | | ^5^ | |  |  |  |
| Saraiva et al. 2013 | 17 | 92 | 32 | 100 | 0.48 [0.25, 0.94] | | ^6^ | |  |  |  |
| Vahabi et al. 2018 | 40 | 112 | 76 | 254 | 1.30 [0.81, 2.08] | | ^7^ | |  |  |  |
| Zacarias et al. 2015 | 81 | 99 | 64 | 75 | 0.77 [0.34, 1.75] | | ^8^ | |  |  |  |
| Total (95% CI) |  | 921 |  | 867 | 1.40 [0.76, 2.59] | |  | |  |  |  |
| Total events | 386 |  | 320 |  |  | |  | | | |  |

References

1. da Silva FRP, Pessoa LDS, Vasconcelos A, de Aquino Lima W, Alves EHP, Vasconcelos DFP. Polymorphisms in interleukins 17A and 17F genes and periodontitis: results from a meta-analysis. *Mol Biol Rep*. Dec 2017;44(6):443-453. doi:10.1007/s11033-017-4128-x

2. Sasikumar PK, Varghese SS, Kumaran T, Devi SS. Meta-Analysis of Risk Association between Interleukin-17A Gene Polymorphism and Chronic Periodontitis. *Contemp Clin Dent*. Jan-Mar 2020;11(1):3-9. doi:10.4103/ccd.ccd_448_19

3. Borilova Linhartova P, Kastovsky J, Lucanova S, et al. Interleukin-17A Gene Variability in Patients with Type 1 Diabetes Mellitus and Chronic Periodontitis: Its Correlation with IL-17 Levels and the Occurrence of Periodontopathic Bacteria. *Mediators Inflamm*. 2016;2016:2979846. doi:10.1155/2016/2979846

4. Chaudhari HL, Warad S, Ashok N, Baroudi K, Tarakji B. Association of Interleukin-17 polymorphism (-197G/A) in chronic and localized aggressive periodontitis. *Braz Oral Res*. 2016;30doi:10.1590/1807-3107BOR-2016.vol30.0026

5. Corrêa JD, Madeira MFM, Resende RG, et al. Association between polymorphisms in interleukin-17A and-17F genes and chronic periodontal disease. *Mediators of inflammation*. 2012;2012

6. Saraiva AM, Alves e Silva MR, Correia Silva Jde F, et al. Evaluation of IL17A expression and of IL17A, IL17F and IL23R gene polymorphisms in Brazilian individuals with periodontitis. *Hum Immunol*. Feb 2013;74(2):207-14. doi:10.1016/j.humimm.2012.10.026

7. Vahabi S, Nazemisalman B, Hosseinpour S, Salavitabar S, Aziz A. Interleukin‐2,‐16, and‐17 gene polymorphisms in Iranian patients with chronic periodontitis. *Journal of investigative and clinical dentistry*. 2018;9(2):e12319.

8. Zacarias JM, Sippert EA, Tsuneto PY, Visentainer JE, de Oliveira e Silva C, Sell AM. The Influence of Interleukin 17A and IL17F Polymorphisms on Chronic Periodontitis Disease in Brazilian Patients. *Mediators Inflamm*. 2015;2015:147056. doi:10.1155/2015/147056

9. Erdemir EO, Hendek MK, Kocakap DBS, Ozkan SY. Interleukin (IL)-17F (H161R) and IL-23R (R381Q) gene polymorphisms in Turkish population with periodontitis. 2015;

10. Jain N, Joseph R, Balan S, Arun R, Banerjee M. Association of interleukin-4 and interleukin-17F polymorphisms in periodontitis in Dravidian ethnicity. *Indian J Hum Genet*. Jan 2013;19(1):58-64. doi:10.4103/0971-6866.112891
