# Supplementary material for: A Systematic Review and Meta-Analysis on Multiple Cytokine Gene Polymorphisms in the Pathogenesis of Periodontitis
Source: Front Immunol. 2022 Jan 3;12:713198. doi: 10.3389/fimmu.2021.713198 (PMC8761621; doi:10.3389/fimmu.2021.713198)
Supplement: Supplementary file 12 [file Table_12.docx]

Table S12. The association of TGF beta rs1800469 polymorphism with chronic periodontitis

|  | case/control | |  | |  | |  | |  | |  |  |
| --- | --- | --- | --- | --- | --- | --- | --- | --- | --- | --- | --- | --- |
| de Souza et al. 2003 | 50/37 | 0.68 | | [0.37, | | 1.25] | | ^2^ | |  |  |  |
| Komatsu et al. 2008 | 113/108 | 1.03 | | [0.71, | | 1.49] | | ^3^ | |  |  |  |
| Kobayashi-a et al. 2009 | 117/108 | 1.09 | | [0.73, | | 1.63] | | ^4^ | |  |  |  |
| Heidari et al. 2013 | 100/100 | 1.11 | | [0.84, | | 1.47] | | ^5^ | |  |  |  |
| Kobayashi-b et al. 2009 | 319/303 | 1.32 | | [0.92, | | 1.89] | | ^6^ | |  |  |  |
| Holla et al. 2002 | 98/108 | 1.35 | | [0.88, | | 2.07] | | ^7^ | |  |  |  |
| Zhao et al. 2010 | 102/102 | 1.72 | | [1.16, | | 2.55] | | ^8^ | |  |  |  |
| Arab et al., 2012 | 24/26 | 2.17 | | [0.99, | | 4.76] | | ^9^ | |  |  |  |

**References**

1. Cui L, Sun YP, Li DG, Wang SH, Shao D. Transforming growth factor-beta1 rs1800469 polymorphism and periodontitis risk: a meta-analysis. *Int J Clin Exp Med*. 2015;8(9):15569-74.

2. de Souza AP, Trevilatto PC, Scarel-Caminaga RM, de Brito RB, Line SR. Analysis of the TGF-beta1 promoter polymorphism (C-509T) in patients with chronic periodontitis. *J Clin Periodontol*. Jun 2003;30(6):519-23. doi:10.1034/j.1600-051x.2003.00323.x

3. Komatsu Y, Galicia JC, Kobayashi T, Yamazaki K, Yoshie H. Association of interleukin-1 receptor antagonist +2018 gene polymorphism with Japanese chronic periodontitis patients using a novel genotyping method. *Int J Immunogenet*. Apr 2008;35(2):165-70. doi:10.1111/j.1744-313X.2008.00757.x

4. Kobayashi T, Murasawa A, Ito S, et al. Cytokine gene polymorphisms associated with rheumatoid arthritis and periodontitis in Japanese adults. *J Periodontol*. May 2009;80(5):792-9. doi:10.1902/jop.2009.080573

5. Heidari Z, Mahmoudzadeh‐Sagheb H, Hashemi M, Rigi‐Ladiz MA. Quantitative Analysis of Interdental Gingiva in Patients With Chronic Periodontitis and Transforming Growth Factor‐β1 29C/T Gene Polymorphisms. *Journal of periodontology*. 2014;85(2):281-289.

6. Kobayashi T, Ito S, Kuroda T, et al. The interleukin-1 and Fcgamma receptor gene polymorphisms in Japanese patients with rheumatoid arthritis and periodontitis. *J Periodontol*. Dec 2007;78(12):2311-8. doi:10.1902/jop.2007.070136

7. Holla LI, Fassmann A, Benes P, Halabala T, Znojil V. 5 polymorphisms in the transforming growth factor-beta 1 gene (TGF-beta 1) in adult periodontitis. *J Clin Periodontol*. Apr 2002;29(4):336-41. doi:10.1034/j.1600-051x.2002.290409.x

8. Zhao X, Guan Z, Zhang Y. Relationship between transforming growth factor beta-1 gene-509C/T polymorphism and severe chronic periodontitis. *Zhonghua kou qiang yi xue za zhi= Zhonghua kouqiang yixue zazhi= Chinese journal of stomatology*. 2010;45(10):610-613.

9. Arab H, Afshari J, Radvar M, et al. Association between TGF-β1-509 Gene Polymorphism with Aggressive Periodontitis. *International Journal of Genetic Engineering*. 2012;2:33-37.
